# Liquid Biopsy for Molecular Residual Disease Detection and Postoperative Surveillance in Gastric Cancer: Current Evidence and Future Directions

**DOI:** 10.3390/ijms27177697

**Published:** 2026-08-28

**Authors:** Lydia Lazaridou, Kalliopi Vakalou, Alexandra Dimaki, Konstantinos Eleftherios Koumarelas, Konstantinos Zachos, Dimitrios Schizas, Grigorios Christodoulidis

**Affiliations:** 1 Department of General Surgery, University Hospital of Larissa, 41110 Larissa, Greece; lazaridoulydia1@gmail.com (L.L.); vakaloukalliopi@gmail.com (K.V.); demakealexandra@gmail.com (A.D.); konstzachos@gmail.com (K.Z.); 2 Department of General and Orthopaedic Surgery, Spitalverbund Appenzell Ausserrhoden, 9100 Herisau, Switzerland; kostaskoumarelas@gmail.com; 3 Department of First Surgery, National and Kapodistrian University of Athens, 11527 Athens, Greece; schizasad@gmail.com

**Keywords:** gastric cancer, liquid biopsy, circulating tumor DNA, molecular residual disease, minimal residual disease, molecular relapse, circulating tumor cells, extracellular vesicles, exosomes, cell-free RNA, postoperative surveillance

## Abstract

Gastric cancer remains a major cause of cancer mortality worldwide, mainly due to its frequent diagnosis at advanced stages and the high probability of recurrence even after curative treatment. Conventional postoperative follow-up is mainly based on imaging studies, endoscopy and serological tumor markers, such as carcinoembryonic antigen (CEA), carbohydrate antigen 19-9 (CA19-9), and carbohydrate antigen 72-4 (CA72-4)This narrative review was based on a structured literature search of PubMed, Scopus, Web of Science, MEDLINE, the Cochrane Library, and ClinicalTrials.gov from database inception through June 2026, with 75 studies included in the final narrative synthesis. Among these analytes, circulating tumor DNA (ctDNA)currently provides the most mature data for the detection of molecular residual disease and postoperative risk stratification. Although it allows for the early detection of recurrent or residual disease prior to imaging confirmation, postoperative ctDNA has shown significant prognostic value in many studies; however, routine use as a basis for decision-making in treatment planning is still considered investigational and will require future prospective clinical validation. Tumor-informed ctDNA approaches offer high analytical specificity and sensitivity in low-burden disease settings. In contrast, tumor-agnostic approaches, such as methylation analysis and fragmentomics, may improve the scalability of the method. However, they require further validation in the postoperative setting. At the same time, emerging data indicate that extracellular vesicles, exosomal RNA and peritoneal lavage analytes can provide complementary biological information, especially in cases of peritoneal dissemination. Despite the significant prospects, the use of liquid biopsy in guiding the treatment of gastric cancer remains under investigation. This is because even today there are limitations. Characteristic are the low ctDNA excretion and the anatomical heterogeneity of the disease, as well as clonal hematopoiesis. Limitations also include the lack of standardization as well as the cost and the need for prospective clinical studies. This review summarizes the biological basis of molecular residual disease, liquid biopsy technologies, ctDNA data, the concept of molecular recurrence, and the future prospects of multi-analytic and artificial intelligence (AI)-assisted surveillance models in gastric cancer.

## 1. Introduction

Although there has been an overall decline in the incidence of gastric cancer (GC) in some areas of the world, GC remains a significant global health issue due to its aggressive nature and challenges in effectively controlling the disease. Gastric cancer ranks among the top malignancy diagnoses globally and is also among the primary contributors to cancer-related deaths around the world [1,2,3]. The prognosis of gastric cancer depends largely upon the staging of the disease, with a 5-year survival rate for early gastric cancer being greater than 90% compared to less than 20% for those with advanced gastric cancer at the time of diagnosis. Therefore, it is critical to improve methods for detecting gastric cancer earlier and to implement effective surveillance strategies [1,4,5]. Unfortunately, a large number of individuals still present with late-stage gastric cancer, while numerous others who undergo what appears to be curative-intent treatments ultimately experience recurrence and/or death due to the disease [6,7].

The curative-intent management of local gastric cancer has undergone significant changes over recent decades, primarily based on combinations of treatments that include perioperative/adjuvant systemic therapies and radical gastrectomies. Although there have been improvements in the management of localized gastric cancer, recurrence is still the primary reason for treatment failure [8,9]. Even after complete (R0) resections, recurrence can occur frequently in the first few years following surgery and typically presents as unresectable locoregional disease or distant metastasis [10,11,12]. Thus, it would appear that occult micro-metastases and residual malignant clones exist despite apparent removal of all macroscopic disease, and therefore, conventional pathologic/radiologic evaluation cannot reliably define disease-free status.

Presently, postoperative surveillance primarily involves cross-sectional imaging studies (i.e., computer tomography (CT) scans upper gastrointestinal (GI) endoscopies, and serum tumor marker testing using carcinoembryonic antigen (CEA), carbohydrate antigen 19-9 (CA19-9), and Carbohydrate antigen 72-4 (CA72-4) While each modality has certain advantages, it also has significant limitations. Serum biomarkers do not have adequate sensitivity and specificity to accurately assess recurrent disease. Imaging studies are limited by their inability to detect small amounts of disease/micro-metastases in tissues [13,14,15,16]. In fact, visible lesions on cross-sectional imaging usually indicate sufficient cellular growth to be detected via imaging, and thus molecular/biological dissemination/clonal expansion likely occurs before radiographic visualization [2]. As a result, recurrence is often identified after molecular/biological progression has resulted in clinical manifestation of disease, limiting early intervention opportunities.

Given this disparity between clinical/radiologic/pathologic assessment and molecular residual disease (MRD) detection, there is growing interest in understanding MRD, which represents the presence of occult malignant cells/tumor-derived molecular signals in patients whose clinical/radiologic/pathologic findings indicate no evidence of active disease following curative-intent treatment [13,14,15,16,17,18]. Additionally, it is increasingly recognized that MRD serves as the biological basis for relapse/recurrence and provides a mechanism for explaining how recurrence occurs in patients who appear to be disease-free [11,12,13]. Notably, the time period between molecular recurrence and clinical relapse/recurrence could represent a therapeutic window during which the disease burden is relatively low and potentially easier to treat [12].

Liquid biopsy most commonly refers to an analysis of material derived from tumors in peripheral blood that is minimally invasive. However, markers derived from tumors can also be analyzed in specific regional fluids such as peritoneal lavage fluid, malignant ascites and cerebrospinal fluid under selected clinical circumstances. Unlike plasma liquid biopsy, samples specific to compartments are obtained invasively and are available only under certain clinical circumstances, thus serving to supplement rather than take the place of routine surveillance using blood samples following surgery [19,20]. Liquid biopsies encompass a wide variety of analytes including circulating tumor DNA (ctDNA), circulating tumor cells (CTCs), cell-free RNA, extracellular vesicles/exosomes, and emerging multi-omic molecular signatures detectable in peripheral blood/body fluids [17,18,21]. Of these analytes, ctDNA has received particular attention due to its potential to directly report tumor-specific genetic alterations and to detect residual disease months before recurrence becomes apparent via conventional imaging studies [8,14,19].

In addition to acting as passive biomarkers for monitoring the presence of residual disease, some liquid biopsy analytes (including extracellular vesicles and exosomal nucleic acids) offer insights into the biological processes responsible for tumor progression/metastasis/disease recurrence/treatment resistance, etc. [20,21,22]. Furthermore, advancements in genomics/epigenomics/transcriptomics/fracture-based analysis are rapidly expanding liquid biopsy capabilities beyond mutational detection towards comprehensive characterization of dynamic tumor ecosystems [23,24].

Liquid biopsies offer an opportunity to redefine recurrence in gastric cancer from a radiologic event to a biologic process prior to when it is evident at the clinical level. Here, we discuss how liquid biopsies may be used as a window for residual disease in gastric cancer with a focus on identifying molecular relapse, determining recurrence risk, monitoring postoperative surveillance and potentially enabling earlier therapy. We will review the current literature across ctDNA, CTCs, extracellular vesicles (EVs) and other circulating biomarker(s), in order to determine if molecular detection can bridge the gap between the clinically undetectable residual disease and clinical reality.

To improve clinical interpretation we organize evidence according to the specific clinical context where liquid biopsies are used. Differentiating detection of postoperative molecular residual disease from longitudinal surveillance, as well as monitoring response to treatment and early detection, is important because these contexts vary greatly with respect to burden of tumor, shedding of ctDNA, assay performance, sampling strategies and biological goals and clinical endpoints. Accordingly findings from advanced or metastatic disease are discussed separately and are not considered directly applicable for MRD assessment unless specifically noted.

## 2. Materials and Methods

This article is a narrative review. It has been developed from the results of an electronic literature search through the following databases: PubMed, Scopus, Web of Science, MEDLINE, the Cochrane Library and ClinicalTrials.gov. All articles identified as having addressed the topic of liquid biopsy in gastric cancer since each database’s inception through June 2026 have been considered. Articles addressing ctDNA, CTCs, EVs, ,cell-free RNA (cfRNA) MRD, molecular relapse, postoperative surveillance and emerging biomarker-guided management approaches will be considered.

A combination of Medical Subject Heading (MeSH) terms and free text keyword searching utilizing the Boolean operators AND and OR was utilized within all searches. Keywords used in these searches include: Gastric Cancer; Gastric Adenocarcinoma; Gastroesophageal Junction Cancer; Liquid Biopsy; Circulating Tumor DNA; ctDNA; Cell-Free DNA; cfDNA; Circulating Tumor Cells; Extracellular Vesicles; Cell-Free RNA; MicroRNA; Long Non-Coding RNA; Molecular Residual Disease; Molecular Relapse; Postoperative Surveillance; and MRD-Guided Therapy.

Studies that met the inclusion criteria of relevance to the objectives of this review were selected for further analysis. Preference was given to studies including original data, systematic reviews, meta-analyses and landmark clinical trials assessing both the biologic rationale and diagnostic/prognostic/clinical utility of liquid biopsy in gastric cancer. Studies reported as conference abstracts with insufficient methodology detail, non-English language publications, animal models and those irrelevant to gastric cancer were typically excluded. In addition, reference listings of selected relevant publications were examined for identification of other pertinent studies.

## 3. Biology of Minimal Residual Disease

### 3.1. Definition of MRD

Minimal residual disease (MRD)—now generally referred to as molecular residual disease (MRD) due to the fact that it is measured using molecular techniques—refers to the microscopic presence of cancerous cells or molecular components within a patient after treatment for potentially curable cancer, while there is no visible cancer or radiographic evidence of cancer that could be detected via standard staging methods [1,2,10,25]. The remnants include circulating tumor DNA (ctDNA), circulating tumor cells (CTCs), non-coding RNA, and exosomes found in either peripheral blood or lavage fluid from the peritoneum before the first signs of recurrence appear [7,9,11]. Detection of tumor-specific ctDNA containing somatic single nucleotide variations, copy number alterations, microsatellite instability, or abnormal methylation patterns within this window constitute formal molecular evidence of MRD and identify those at high risk of developing clinical recurrence [26,27].

The term molecular residual disease (MRD) is preferred over minimal residual disease because this is current terminology in solid tumor oncology. MRD refers to the persistence of residual malignant cells or molecular signals derived from tumors that treatment intended to be curative cannot detect using clinical, radiological or endoscopic assessment but can be detected using very sensitive molecular assays, especially technologies based on circulating tumor DNA. Τhus, MRD represents a detectable molecular state that comes before clinically evident recurrence of disease. Postoperative positivity for ctDNA represents molecular evidence of MRD when tumor DNA is detected following curative surgery. On the other hand, molecular relapse or recurrence refers to subsequent detection or reappearance of ctDNA during longitudinal surveillance following an initial assessment before clinical or radiological recurrence occurs. Simultaneously, ctDNA describes the biological release of tumor DNA into circulation and depends on tumor burden, vascularization, and metastatic site, while clearance of ctDNA refers to disappearance of previously detectable ctDNA following treatment and indicates a molecular response [7,11,14,28].

Detection of ctDNA is analytically challenging because only <0.01% of cfDNA in plasma comes from tumors and only about 0.014% of a tumor cell’s DNA is typically shed into circulation under normal physiological conditions [28]. The quantity of measurable ctDNA corresponds to tumor size and extent of metastasis, and thus liver and lung metastases generate much larger shedding rates compared with lymph nodes or peritoneal spread—an important biological discrepancy with direct implications for MRD assay sensitivity in gastric cancer, in which peritoneal dissemination is predominant [28,29]. Moreover, achieving near-total pathological regression of the primary site does not necessarily mean that systemic disease was biologically eliminated; some patients with significant pathological regression can still harbor measurable amounts of ctDNA postoperatively and subsequently develop recurrence, demonstrating that morphological assessments are inadequate for detecting residual tumor burden [12,30].

### 3.2. Mechanisms of Recurrence

The biological basis for the recurrence of tumors after the apparent cure of a patient with potentially curative surgery involves remnants of very small amounts of microscopic tumor that have been able to avoid detection by current diagnostic techniques. Cells from primary lesions can break off and be distributed throughout the body either by direct entry into blood vessels or by migration to bone marrow during the initial phases of cancer development, where they may remain dormant for an extended period (e.g., months to years) before undergoing active proliferation [2,9].

Cells responsible for producing circulating molecular signals in cases of minimal residual disease (MRD) produce these signals through ongoing apoptosis and necrosis in microscopic tumor aggregates that release fragmented nucleic acids directly into the vascular space. These same tumor cells also actively secrete DNA-containing microvesicles (exosomes), providing additional means of producing and releasing these signals [1,28].

Residual disease in the peritoneum—the most common site of recurrence in patients with gastric cancer—occurs according to the “seed–soil” model. Cancer cells detach from the original tumor, penetrate the serosa, enter the abdominal cavity as free intraperitoneal cells, attach to mesothelial cells lining the peritoneum, and invade the subjacent subperitoneal connective tissue to create micro-metastases [25,31]. Furthermore, pre-metastatic peritoneal microenvironments are created in advance through exosome-mediated mesothelial–mesenchymal transition (MMT). In this process, tumor-derived exosomes induce transformation of the peritoneal stroma into a fibrotic environment conducive to micrometastasis formation from free intraperitoneal cells, well before any detectable disease appears on currently available imaging technologies [32].

Compared to liver and lung metastases, peritoneal implants are less well perfused and often confined to the peritoneal space, limiting the amount of tumor-derived DNA released into the peripheral blood. Therefore, individuals who present with isolated peritoneal dissemination typically have lower levels of circulating free tumor DNA (ctDNA) in their plasma, which can reduce assay sensitivity and potentially result in false-negative plasma test results [3,7,33]. The fact that tumors confined to the peritoneum are limited to the peritoneal space suggests there may be additional potential sources for assessing residual disease in the metastatic niche of the peritoneum such as through examination of peritoneal lavage fluid or malignant ascites. Several primary studies have investigated molecular analysis of peritoneal lavage fluid as a regional liquid biopsy approach for detecting occult peritoneal disease and predicting subsequent peritoneal metastasis in gastric cancer, as summarized in Table 1. Additionally, because both extracellular vesicles and exosomal RNA contain information about residual disease within these compartments, they may provide alternative methods for detecting residual disease in the metastatic niche of the peritoneum [32,33].

Peritoneal dissemination represents a biologically distinct pattern of metastatic spread and is now recognized as a specialized metastatic niche rather than merely an anatomical site of recurrence. Successful peritoneal colonization depends on dynamic interactions between tumor cells and the local microenvironment, resulting in biological divergence from the primary gastric tumor and the establishment of a permissive metastatic ecosystem [32,33].

Mesothelial cells play a central role in this process by undergoing mesothelial-to-mesenchymal transition (MMT) after being exposed to tumor-derived cytokines, transforming growth factor-β (TGF-β), and extracellular vesicles. As a result of this phenotypic transformation, mesothelial cells undergo changes that promote extracellular matrix remodeling, fibroblast activation, increased tumor cell adhesion, and invasion into the submesothelial connective tissue. All of these processes facilitate the successful implantation of metastatic tumor cells [20,32,33]. Simultaneously, cancer-associated fibroblasts remodel the extracellular matrix and produce growth factors that stimulate tumor proliferation, angiogenesis, and resistance to chemotherapy, whereas tumor-derived exosomes enhance stromal remodeling and intercellular communication in the metastatic environment [6,20,21,32,33].

The immune composition of the peritoneal microenvironment differs significantly from that found at the primary tumor site. An increase in M2-polarized macrophages, regulatory T cells, myeloid-derived suppressor cells, and immunosuppressive cytokines supports immune escape mechanisms and contributes to the prolonged survival of disseminated tumor cells [6,20,21,22,33]. Moreover, malignant ascites acts as a biologically active compartment containing tumor cells, extracellular vesicles, cytokines, and cell-free nucleic acids. These components continuously interact with stromal and immune cells located in the metastatic site to facilitate continued metastasis and resistance to treatment [32,33].

These biological features have significant implications for liquid biopsy. Because of their relative lack of vascularity and frequent confinement to the peritoneal space, peritoneal implants generally do not freely release tumor-derived DNA into the peripheral blood. As a consequence, many patients with isolated peritoneal dissemination have low levels of ctDNA in their plasma even though their tumor burden is extensive. Low plasma ctDNA levels decrease assay sensitivity and increase the likelihood that plasma-based tests will yield false-negative results [3,7,33]. Furthermore, because of this compartmentalization, it is reasonable to investigate complementary biomarkers derived from other sources such as malignant ascites, extracellular vesicles, exosomal RNAs, and/or peritoneal lavage fluids. It is possible these biomarkers could serve as surrogate indicators of residual disease that exists in the peritoneal metastatic niche [32,33].

Recurrence does not occur solely due to persistence of microscopic tumor cells, but rather results from two-way communication between microscopic tumor deposits and their local microenvironment. Microscopic tumor cells communicate through tumor-derived exosomes that carry oncogenic cargo (i.e., mRNAs, miRNAs, proteins, and lipids) to recipient stromal and immune cells within the tumor niche, resulting in activation of processes such as epithelial-to-mesenchymal transition (EMT), neovascularization, suppression of host immunity, and resistance to chemotherapy [6,34]. Through the transfer of let-7g-5p via exosomes, tumor cells promote polarization of recipient M2 macrophages that suppress immune responses against occult clones of malignant cells, whereas exosomal PD-L1 induces expansion of myeloid-derived suppressor cells (MDSCs) through activation of the IL-6/STAT3 pathway, thereby suppressing immune function against tumors [20,22]. Together, these mechanisms demonstrate that recurrence post-gastrectomy represents a self-sustaining ecosystem based on mutualism between tumor cells and their respective microenvironments, rather than merely passive survival of undetected tumor cells [16].

**Table 1:** Representative primary studies evaluating peritoneal lavage based liquid biopsy in gastric cancer.

Study (Ref.)	Study Design and Population	Tumor Stage/Setting	Sampling Time and Specimen	Biomarker/Assay	Clinical Application	Main Findings	Major Limitations
Yukawa et al. [31]	Feasibility study; 15 patients with gastric cancer	Patients undergoing surgery; included cytology-positive and cytology-negative cases	Intraoperative lavage samples obtained from the Douglas pouch and left subdiaphragmatic area	Peritoneal tumor DNA; cell-free DNA extraction followed by ddPCR detection of *TP53* mutations	Molecular detection of free intraperitoneal tumor cells and prognostic assessment	Peritoneal tumor DNA was detected in 6 of 10 cytology-positive patients in Douglas pouch samples and in none of the five cytology-negative patients. Peritoneal tumor DNA positivity was associated with shorter overall survival. The molecular approach showed diagnostic utility comparable to conventional microscopic cytology.	Very small exploratory cohort; analysis restricted mainly to TP53 mutations; sampling-site variation; no external validation.
Bai et al. [35]	Prospective single-center study of patients with stage III gastric cancer	High-risk patients undergoing radical resection	Peritoneal lavage fluid collected before and after resection	PLF ctDNA analyzed by NGS; CTCs detected using EpCAM-, folate receptor- and cytokeratin-based immunofluorescence	Prediction of metachronous peritoneal metastasis after surgery	Preoperative and postoperative ctDNA positivity were associated with increased peritoneal-metastasis risk. Postoperative ctDNA produced an AUC of 0.93, compared with 0.86 for preoperative ctDNA. Combined postoperative ctDNA/CTC positivity identified the highest-risk group, with HR 18.14 (95% CI 3.27–100.70), and the combined approach achieved 85% accuracy.	Single-center study; relatively limited cohort and number of recurrence events; wide confidence intervals; assay thresholds require external validation; not yet suitable for directing treatment outside clinical studies.
Yue et al. [36]	Multi-cohort validation study; exploratory cohort of 104 patients plus an independent validation cohort of 76 patients; total n = 180	Gastric cancer patients evaluated for subsequent peritoneal metastasis	Pre-resection and post-resection peritoneal lavage fluid; matched plasma ctDNA; serial sampling in five patients receiving intraperitoneal chemotherapy	Personalized mutation profiling to estimate cancer-cell fraction in PLF	Early prediction of peritoneal metastasis, postoperative risk stratification and exploratory treatment-response monitoring	Pre-resection PLF cancer-cell fraction demonstrated 98% sensitivity and 80% specificity, whereas post-resection PLF demonstrated 82% sensitivity and 90% specificity. Combining pre- and post-resection results achieved 100% sensitivity and 80% specificity. PLF cancer-cell fraction was a stronger predictor of peritoneal metastasis than conventional lavage cytology or plasma ctDNA.	Despite multi-cohort validation, broader prospective external validation is required; personalized mutation profiling may increase cost and complexity; treatment monitoring was evaluated in only five patients; clinical utility for selecting intraperitoneal treatment remains investigational.

### 3.3. Tumor Heterogeneity and Clonal Evolution

The genetic diversity found within tumors is one of the biggest biological obstacles to effectively eradicating microscopic amounts of cancer and achieving long-lasting clinical benefits with therapies [37]. The reason for this is because a tissue biopsy from one site on the body captures only the genes at that location. It will miss any other unique gene abnormalities present in tumor cells in a different area of the body. Studies using DNA sequencing from many parts of the same tumor have shown that about 50 percent of all the changes in DNA that occur in cancers such as gastric cancer can vary in their distribution among the various tumor cells [30]. Therefore, what appears to be a complete picture of a patient’s cancer based upon analysis of a small portion of the tumor can significantly under-represent how much genetic damage is being produced by those cancerous cells that lead to recurrence after treatment.

When analyzing two or more genetically diverse areas of a single tumor (such as the original site vs. a metastasis), there can also be significant differences in the degree to which each region contains specific high-risk amplification regions of DNA, including amplifications of the *HER2* gene, the *EGFR* gene, the *CDK4/6* gene and the *MET* gene. These types of variations are especially common in tumors that have been treated with drugs and are therefore often difficult to treat.

Cancer cells exist within a microenvironment where they constantly undergo genetic evolution through random errors in DNA replication and repair and/or selective pressure from treatments [38]. Based on our understanding of the clonal evolution hypothesis, systemic treatments selectively kill off sensitive cancer stem cells while allowing previously existing drug-resistant clones to grow back into the predominant population responsible for future recurrences [39]. For example, researchers have used longitudinal liquid biopsies to show that plasma levels of circulating tumor DNA (ctDNA) corresponding to HER2-positive gastric cancer progressively become dominated by clones that do not carry additional copies of the HER2 gene during administration of anti-HER2 therapies [30]. Furthermore, these data suggest that the genetic characteristics of recurring cancer reflect clonal selection rather than simply persistence of the parental clone [8,9]. Low-frequency resistance alleles can be identified at the time of first diagnosis in some cases and appear to disappear from plasma after effective therapy, only to reappear later in time before clinical evidence of recurrence becomes evident. Although it may be theoretically possible to identify pre-existing resistance alleles using deep sequencing technologies, it is unlikely that such methods would be practical for routine clinical application [40].

In order to accurately assess whether a particular mutation occurs throughout a large proportion of a tumor or if it is limited to a smaller subset of cancer cells, it is necessary to convert the measured frequency of mutant alleles in plasma into an estimate of the number of cancer cells per unit volume of blood. Truncal clonal events represent mutations that occur in every cell in a tumor, while mutations that occur in less than 100% of cancer cells represent subclonal events [30]. Subclonal mutations may individually contribute to treatment resistance. Because tracking the entire spectrum of subclonal mutations occurring in tumors is critical for identifying potential mechanisms contributing to the failure of targeted therapeutics, population-based multi-gene tracking strategies examining panels of tumor-informed somatic variants detected in serial plasma samples provide more comprehensive views of evolving subclonal landscapes than single-mutation tracking approaches [9]. By pooling DNA released from multiple anatomical locations into a single sample, liquid biopsy provides a real-time, unbiased assessment of a tumor’s global genomic architecture [9]. The advantage provided by liquid biopsy is further amplified by its ability to monitor complementary biological information provided by nucleic acids (ctDNA and ccfRNA): while ctDNA provides somatic mutational and epigenetic signatures derived from dying and dead cancer cells, ccfRNA captures dynamically changing expression patterns emanating from neoplastic and surrounding stromal components [17,41]. Detection and quantitation of minimal residual disease (MRD) should therefore be viewed as a dynamic, ongoing process of subclonal expansion and evolution in which the period between measurable presence in plasma and appearance as clinically relevant radiographic findings—possibly extending for months—defines the most timely and actionable opportunity for therapeutic intervention [30].

Tumor heterogeneity in gastric cancer extends beyond alterations in the genome and also includes important phenotypic and lineage-specific differences that impact biology and patterns of disease dissemination [30,37,42]. Gastric adenocarcinomas show distinct differentiation programs of gastric and intestinal types, as well as variable mucin expression profiles and differences in lineage-associated markers, reflecting diverse biological states [43]. Emerging evidence suggests that these phenotypic features may be associated with different routes of metastasis, including preferential dissemination to the peritoneum or via the blood, which may influence the quantity and composition of tumor material released into circulation. Biomarker performance, including the detectability of ctDNA and other liquid biopsy analytes, therefore, may differ not just by burden alone [28,29]. Novel lineage-associated biomarkers such as VSIG1 have also highlighted the biological diversity of gastric cancer by identifying distinct molecular and phenotypic subgroups with potential prognostic significance and different metastatic behavior. Collectively, these observations support the idea that comprehensive characterization of gastric cancer should integrate both genomic and phenotypic heterogeneity, providing a more complete framework for interpreting findings from liquid biopsies and for future personalized surveillance development [37,43].

## 4. Liquid Biopsy Technologies

Liquid biopsy in clinical oncology refers primarily to minimally invasive analysis of material derived from peripheral blood that comes from tumors. While markers derived from tumors can also be evaluated in fluids like peritoneal lavage fluid, ascites that has become malignant and cerebrospinal fluid, these specimens are only collected under selected clinical circumstances and should be considered specific sampling approaches rather than routine systemic liquid biopsies. In this way, it offers a dynamic alternative or complementary method to conventional tissue biopsy in gastric cancer. In contrast to tissue biopsy, which is usually limited to a specific site of the tumor, liquid biopsy can better capture the spatial and temporal heterogeneity of the disease. It can also be repeated during treatment as well as during postoperative follow-up and long-term surveillance. The main analytical components of liquid biopsy include circulating tumor DNA (ctDNA), circulating tumor cells (CTCs) and exosomes. In addition, they include extracellular vesicles (EVs) and various forms of cell-free RNA (cfRNA). Typical examples are microRNAs, long non-coding RNAs, and circular RNAs, as well as other small non-coding RNAs. Each technology provides a different level of biological information. More specifically, ctDNA mainly reflects the genomic and epigenomic alterations of the tumor, whereas CTCs retain the cellular and phenotypic characteristics of living cancer cells. In contrast, EVs and cfRNAs can reveal functional processes related to tumor–microenvironment communication, immune evasion, metastatic capacity, and resistance to therapy [2,37,44].

### 4.1. Circulating Tumor DNA

Cell-free DNA (cfDNA) is a collection of broken-down extracellular DNA that circulates throughout the body as a result of both physiologic and pathologic cell death, including apoptosis, necrosis, and active secretion. Circulating tumor DNA (ctDNA) is a subset of the total amount of cfDNA which represents the genetic material present within the circulating portion of cfDNA that has been altered by the tumor. Therefore, while cfDNA consists of DNA fragments from both non-cancerous and cancerous cells, ctDNA contains cancer-specific molecular changes, including but not limited to somatic mutations, copy number variations, structural variations and aberrant methylation patterns. As such, cfDNA represents all types of cellular DNA, whereas ctDNA provides specific cancer-related molecular information and serves as the primary liquid biopsy analyte used today to detect molecular residual disease in patients with gastric cancer, monitor recurrence, and provide comprehensive genomic profiling [9,10,11,44].

ctDNA analysis can be performed with highly sensitive technologies. Specifically, next-generation sequencing (NGS), droplet digital PCR and digital PCR can be used. Methylation-specific PCR, Cancer Personalized Profiling by Deep Sequencing(CAAP-Seq)and targeted gene panels can also be used. This allows the detection of rare neoplastic mutations within a large background of non-neoplastic cfDNA [26,38]. In gastric cancer, ctDNA has been used not only to detect mutations but also to identify therapeutically useful alterations. Typical examples include HER2 amplification, copy number changes, and genomic mechanisms of resistance. For example, plasma cfDNA analysis with Guardant360 has been used to detect HER2 amplification and other genomic alterations without relying solely on immunohistochemical evaluation of tissue samples. In contrast, plasma HER2 amplification and an adjusted HER2 copy number have been associated with a response to HER2-targeted therapies [39,45]. These studies were performed predominantly in advanced or metastatic gastric cancer and primarily illustrate the ability of ctDNA to identify actionable genomic alterations. Their findings should not be interpreted as evidence supporting postoperative MRD detection, where biological characteristics and ctDNA shedding differ substantially [39]. Furthermore, newer cfDNA approaches are not limited to mutation detection but incorporate methylation data, fragmentomics, end motifs, copy number variations, repeat landscapes and machine learning models. Methods such as SPOT-MAS, ARTEMIS and GutSeer show the transition from the classical analysis of single mutations to a multidimensional exploitation of cfDNA signals. In this case, cancer patterns can be detected even when tumor-specific mutations are difficult to identify [18,23,46]. In the context of minimal residual disease (MRD), ctDNA is of particular interest. This is because its sequential analysis can reveal molecular residual disease or molecular relapse before imaging or clinical confirmation. However, its analytical performance may be reduced in low-burden disease settings, particularly in early-stage tumors and isolated peritoneal metastases [7,47]. These approaches are primarily being developed for early cancer detection and population-level screening rather than postoperative molecular residual disease surveillance [23]. The primary research to evaluate liquid biopsy use in early gastric cancer detection, as well as population-level screening, has been reviewed (Table 2). In contrast to the postoperative MRD studies that evaluated the relationship of MRD with recurrence, this group of studies focused on the evaluation of the diagnostic accuracy of liquid biopsies by assessing sensitivity, specificity, Area Under Curve from Receiver Operating Characteristic area under the curve and Early-Stage Detection Rates.

### 4.2. Circulating Tumor Cells

Circulating tumor cells are intact malignant cells that are detached from the primary tumor or metastatic foci and enter the bloodstream. ctDNA consists of fragmented nucleic acids. In contrast, CTCs maintain cell morphology and viability, as well as genomic information and surface protein expression. This allows for simultaneous molecular and phenotypic analysis of cancer cells [2,49]. In gastric cancer, CTCs are particularly important, as they can provide information on hematogenous dissemination, epithelial–mesenchymal transition, the existence of subpopulations with stem cell characteristics and the development of therapeutic resistance. Their detection is based on physical or biological enrichment techniques. Typical examples include density gradient centrifugation, as well as size-based filtration and microfluidic platforms. Immunomagnetic capture and positive selection with epithelial markers such as epithelial cell adhesion molecule(EpCAM)and cytokeratins, as well as negative selection with leukocyte markers such as CD45 have also been observed [50,51]. Commercial and research platforms, such as CellSearch and, Subtraction Enrichment and Immunostaining-Fluorescene In Situ Hubridization(SE-Ifish) have improved the detection of CTCs. In contrast, techniques based FISHon cell size and deformability can help identify mesenchymal or EpCAM-low CTCs. However, the clinical use of CTCs remains technically challenging. This is because they are extremely rare cells, often only a few per milliliter of blood, with a short biological half-life. In addition, they can be destroyed by mechanical forces in the circulation, eliminated by the immune system, or lose epithelial markers during epithelial–mesenchymal transition. This increases the risk of false-negative results [49,51].

### 4.3. Extracellular Vesicles and Small Extracellular Vesicles

Extracellular vesicles (EVs) comprise a heterogeneous population of membrane-bound particles that differ in their cellular origin, size, and biogenesis. In accordance with the recommendations of the International Society for Extracellular Vesicles (MISEV), the broader terms “extracellular vesicles” or “small extracellular vesicles (sEVs)” are preferred unless the endosomal origin of the isolated vesicles has been experimentally confirmed. Exosomes and extracellular vesicles are lipid bilayer-encapsulated nanovesicles. Specifically, they are usually about 30–150 nm in size and are secreted by cancer cells and immune cells, as well as fibroblasts. They can also be secreted by endothelial cells, macrophages, neutrophils, and other cells of the tumor microenvironment. They contain and protect biological cargoes such as DNA, proteins, lipids, microRNAs, long non-coding RNAs, circular RNAs, and other regulatory molecules. Typical vesicle-associated markers are CD9, CD63, CD81, TSG101, ALIX and HSP70. These proteins help to identify small extracellular vesicles but do not independently confirm an endosomal origin and therefore cannot be used to definitively distinguish exosomes from other extracellular vesicle populations. They can also play a crucial role in immune evasion, angiogenesis, epithelial–mesenchymal transition, and resistance to therapy. For example, exosomes derived from gastric cancer cells can promote the polarization of macrophages towards an M2 phenotype. Extracellular vesicles are not only passive biomarkers but also active mediators of disease progression, metastasis, immune evasion, angiogenesis, epithelial–mesenchymal transition and therapeutic resistance in gastric cancer. They can also enhance the migration and invasion of cancer cells and remodel the immune microenvironment [20,21]. Exosomal let-7g-5p has been linked to SERPINE1-induced macrophage polarization via the SOCS7/STAT3 axis. In contrast, N2 neutrophil-derived exosomes carrying miR-4745-5p and miR-3911 promote the migration, invasion, proliferation, epithelial–mesenchymal transition, and metastatic potential of gastric cancer cells through the suppression of SLIT2 [20,52]. Exosomal circular RNAs are also emerging as clinically interesting molecules. For example, plasma exosomal circPTBP3 has been associated with peritoneal metastasis of gastric cancer. It has also shown diagnostic value for detecting peritoneal metastatic disease and has been associated with worse overall survival [32]. In addition, exosomal approaches include detecting the gastric cancer-associated lncRNA-GC1 in extracellular vesicles as well as non-molecular techniques. Typical examples include analyzing exosomes with surface-enhanced Raman spectroscopy combined with artificial intelligence. This shows that EVs can be used in both molecular and spectroscopic liquid biopsy platforms [53,54]. Despite the wealth of biological information they convey, exosomal biomarkers remain less standardized than ctDNA technologies. This is mainly due to heterogeneity in isolation methods, characterization protocols, and marker selection and clinical validation (Table 3).

### 4.4. Cell-Free RNA and microRNAs

Cell-free RNA includes a wide range of circulating RNA molecules, such as microRNAs, long non-coding RNAs, circular RNAs, PIWI-interacting RNAs, transfer RNA-derived small RNAs, ribosomal RNA-derived small RNAs and other regulatory RNA fragments. These molecules can be released through apoptosis, necrosis, and active secretion or transport via extracellular vesicles. However, many are stabilized in circulation because they are encapsulated in exosomes or EVs [41,51]. MicroRNAs are small non-coding RNAs of approximately 21–24 nucleotides in length. They regulate gene expression at the post-transcriptional level and can function as either oncogenes or tumor suppressor molecules. In gastric cancer, circulating and exosomal microRNAs have been implicated in cell proliferation as well as invasion and metastasis. They have also been implicated in immunoregulation, epithelial–mesenchymal transition, peritoneal dissemination, and resistance to chemotherapy [59,60]. For example, exosomal miR-21-5p has been associated with peritoneal metastasis via mesothelial–mesenchymal transition. Conversely, macrophage-derived exosomal miR-21 may contribute to cisplatin resistance [59]. In addition to classical microRNAs, EV-derived small non-coding RNAs are increasingly recognized as promising liquid biopsy biomarkers. Analysis of plasma EV small RNAs with PANDORA-seq in gastric cancer revealed multiple RNA classes. Typical examples include miRNAs, piRNAs, and ribosomal RNA-derived small RNAs, as well as transfer RNA-derived small RNAs and snoRNAs. In contrast, a triple rs/tsRNA signature showed high diagnostic performance and outperformed conventional protein markers in comparisons with machine learning models [58]. Circular RNAs are also of particular interest. Their circular structure makes them more stable and often enriched in exosomes. Examples such as circATP8A1 and circPTBP3 show that exosomal circRNAs can reflect tumor progression, metastatic behavior, and tumor–microenvironment interactions [32,56]. Although cfRNA and microRNA analyses can capture functional aspects of tumor activity more directly than ctDNA, their clinical application is limited by several factors. Typical examples include the instability of some RNA species, the variability of pre-analytical procedures, as well as the lack of uniform normalization methods and the need for prospective validation. The principal studies evaluating circulating RNA biomarkers in gastric cancer are summarized in Table 4. These studies investigate circulating microRNAs, long non-coding RNAs, circular RNAs and other cell-free RNA species for early diagnosis, recurrence prediction, treatment monitoring and molecular characterization.

### 4.5. Comparative Advantages and Limitations

The main advantage of liquid biopsy is that it is a minimally invasive, reproducible and dynamic method. It is capable of providing real-time information on tumor progression. Compared with tissue biopsy, liquid biopsy can reduce the problem of spatial sampling bias as well as detect molecular changes originating from multiple disease foci and support longitudinal monitoring during treatment and postoperative surveillance [39,61]. ctDNA offers a level of clinical maturity that has made it the best known method for detecting MRD and genomic changes due to its very high analytical specificity and the ability to detect tumor-specific genetic mutations (including resistance), methylation events, chromosomal amplifications/deletions (copy number variations) or other mechanisms of resistance from repeated samples [9,10,11,44].

This is because it can identify mutations, methylation patterns, copy number alterations, and resistance mechanisms with high analytical specificity. CTCs provide complete cellular information, including morphology, protein expression, and viable biology of cancer cells. However, their rarity, short half-life, and dependence on capture techniques limit their reproducibility and broad clinical application [47,49]. Exosomes and EVs are attractive because their lipid bilayer protects their internal cargo from degradation. They are also attractive because they reflect active processes of communication between the tumor and the microenvironment and immune remodeling, as well as the establishment of a metastatic nest and therapeutic resistance. However, methodological heterogeneity and limited standardization continue to restrict their routine clinical implementation. cfRNA and microRNAs can provide complementary functional information, especially when incorporated into multi-analyte models. However, they require improvement in the reproducibility of the methods as well as the establishment of reliable cut-offs and validation in clinically representative populations. In conclusion, no single liquid biopsy marker is sufficient to fully capture the biological complexity of gastric cancer. A combined model incorporating ctDNA, CTCs, EVs, and cfRNA may offer the most comprehensive approach for early detection, molecular recurrence monitoring, risk stratification, and postoperative surveillance. However, large prospective studies are still needed before such models can be incorporated into routine clinical practice (Table 5).

Although ctDNA is now the most developed and clinically utilized liquid biopsy analyte for detecting molecular residual disease and for tracking genomic changes over time within patients with cancer, ctDNA does not account for many aspects of gastric cancer’s biology. Each type of circulating analyte captures different types of information about cancer biology that reflect distinct facets of tumor progression or metastasis and/or how a patient responds to therapy.

In comparison to ctDNA, which typically consists of fragmented DNA released from dying cancer cells, circulating tumor cells (CTCs) represent live cancer cells that have been shed into the circulation. Therefore, unlike DNA-based biomarkers, CTCs can simultaneously be characterized at multiple levels (genomically, transcriptomically, proteomically and functionally). As such, CTCs are particularly useful for investigating whether a particular tumor has undergone an EMT program, if the tumor has acquired the ability to form metastases and/or how a tumor acquires drug resistance. However, due to their very low concentration in blood samples, phenotypic heterogeneity among CTC populations, and requirement for various methods to isolate them from normal blood cells, CTC analysis continues to suffer from poor analytical reproducibility and limited potential for widespread use in clinical settings [2,14,16,47,49,50,51].

Unlike ctDNA, which primarily reflects cellular apoptosis associated with tumor growth and metastasis, extracellular vesicles (EVs), including exosomes, do not passively report the status of tumors. Instead, EVs are able to interact with recipient cells and play an active role in regulating signaling pathways that control tumor growth and metastasis. The lipid bilayer membrane of EVs also protects the nucleic acid and protein cargo contained within them from nuclease-mediated degradation, thereby allowing investigators to characterize the complex molecular interactions between tumors and their local microenvironments. Despite these advantages, there are still several technical challenges to overcome prior to widespread clinical implementation. For example, there are significant differences in the methods used to purify EVs from plasma/serum and characterization of EV content, and lack of standards for reporting results across studies [6,20,21,53,62].

Like ctDNA, circulating free RNA (cfRNA), which includes miRNAs, lncRNAs, circRNAs, transfer RNA-derived fragments(tRFs) and other classes of regulatory RNAs that regulate gene expression through post-transcriptional mechanisms, provides biologically meaningful information regarding ongoing transcriptional activities in tumors. Because transcriptional programs often become activated in response to genomic mutations or in response to environmental signals prior to the emergence of corresponding genomic mutations, these types of biomarkers may complement ctDNA-based analyses by indicating when a tumor is undergoing active biological processes like EMT, immunosuppression, or developing resistance to targeted therapies. Like ctDNA however, cfRNA presents several practical obstacles to its clinical adoption. Examples include the instability of RNA molecules, variable sample collection protocols, normalization issues related to quantifying cfRNA concentrations relative to reference RNAs, and a lack of standardized analytical pipelines [40,49,54,57,58,60].

From a clinical standpoint, the choice of best liquid biopsy analyte is highly dependent upon the specific application being considered. ctDNA has demonstrated the greatest body of evidence supporting its utility in detecting postoperative minimal residual disease, predicting recurrence risk, and longitudinally assessing genomic changes [8,19]. While CTCs may provide some additional prognostic value and/or enable investigators to functionally assess viable tumor cells using techniques such as flow cytometry, EV-derived biomarkers and cfRNA appear to offer the greatest promise for examining the relationship between tumors and their local environments, diffuse peritoneal carcinomatosis, and mechanisms underlying therapeutic resistance [6,40,47]. Therefore, these analytes should be viewed as complementary rather than competitive biomarkers [49].

Therefore, future liquid biopsy assays are likely to utilize multiple analytes, including but not limited to ctDNA, CTCs, EV-derived biomarkers, cfRNA, methylation profiling, fragmentomics, and AI-driven computational models as part of multi-analyte platforms designed to identify both the genomic architecture of residual disease and the functional properties of remaining cancer cells [17,49]. These integrated approaches could potentially increase sensitivity for detecting molecular recurrences while addressing the limitations present with each individual biomarker. Nonetheless, prospective clinical validation of these multi-analyte platforms along with development of standardized methods for measuring these analytes remains essential prior to routine clinical use [49].

Although the current data on ctDNA represent a significantly more developed state than any other type of liquid biopsy assay that can be used to monitor patients after surgery, there remains considerable heterogeneity in the body of literature. The primary source of this heterogeneity is the significant number of different approaches (assay platforms, sequencing depth, threshold of variant calling, when in relation to time of surgery the blood sample was taken, how molecular residual disease is defined) that have been used by researchers [2,7,14]. It appears as though some or all of these variables contribute to the difficulty experienced by investigators in making direct comparisons across studies [3]. This would also appear to help explain the discrepancies observed in sensitivities and lead times of ctDNA detection studies. Additionally, with very few exceptions, most existing research studies investigating the use of ctDNA for monitoring patients after surgery are either retrospective or observational and contain relatively small groups of patients [7,10,14]. As such, although it has become clear through recent studies that the analytical validity of detecting ctDNA continues to increase in quality, the ability to demonstrate definitive benefit from the results obtained from using ctDNA in guiding treatment decisions in prospective randomized controlled trials has not been achieved [2,7,14]. In summary, no individual liquid biopsy component comprehensively encompasses the biological intricacies of gastric cancer. Future multimodal methodologies that incorporate ctDNA, CTCs, extracellular vesicles, and cfRNA could offer a more holistic framework for molecular monitoring, yet prospective clinical validation is crucial prior to standard application.

## 5. ctDNA for MRD Detection in Gastric Cancer

Because ctDNA is the most validated biomarker for MRD assessment in gastric cancer, subsequent sections focus mainly on its use to identify postoperative molecular residual disease. Importantly, serial monitoring of ctDNA goes beyond just detecting MRD by also identifying molecular relapse which is a distinct event that shows renewed positivity over time during surveillance following initial treatment [7,11,14].

### 5.1. Tumor-Informed Approaches

Tumor-informed ctDNA assays represent the highest analytical rigor approach to monitoring for minimal residual disease (MRD) in gastric cancer. Tumor-informed ctDNA assays generally begin with sequencing of the resected tumor together with a matched normal sample to identify patient-specific somatic alterations. Depending on the assay platform, this may involve whole-exome sequencing or targeted genomic sequencing, after which individualized panels are designed for longitudinal plasma monitoring. The number of patient-specific variants incorporated into these personalized panels varies among assays and studies and should not be considered a fixed characteristic of tumor-informed approaches. Ultra-deep longitudinal plasma sequencing is used to monitor for circulating tumor DNA (ctDNA) using the targeted panels [9,11,19]. Under optimized analytical conditions, some tumor-informed assays have reported limits of detection approaching variant allele frequencies of approximately 0.01%; however, these values represent analytical assay performance under specific experimental conditions and should not be interpreted as equivalent to clinical sensitivity in postoperative MRD detection. Several studies report very high specificity for selected assays informed by tumors when evaluated using matched controls that are normal. However specificity varies depending on assay design, filtering strategies and study population and should not be considered universal at 100% [9,49]. Ultra-deep sequencing is commonly used to improve detection of low-frequency variants in surgical samples that have very low abundance of ctDNA. Depth of sequencing required varies among different platforms, assay designs and pipelines and reported numbers such as 65,000 times are specific to assays rather than universal requirements [11]. An essential component of utilizing tumor-informed ctDNA assays for MRD detection is concordance between formalin-fixed, paraffin-embedded (FFPE) tissuetissue and postoperative plasma samples; if there is high concordance between FFPE tissue and postoperative plasma, those concordant somatic mutations provide a very accurate molecular tracking target [63]. Frequently mutated driver genes in gastric cancer such as *TP53*, *CCND1*, *FGF3*, *FGF4*, and *FGF19* provide the most commonly tracked sites. Data collected during prospective cohort studies of patients enrolled in the PLAGAST clinical trial (NCT02674373) identified a significant decline in baseline ctDNA levels in locally advanced resectable gastric/gastroesophageal junction (GEJ) adenocarcinomas from 69.6 percent to 20 percent in the postoperative MRD window [64]. This study included patients with gastric and gastroesophageal junction adenocarcinoma. It also underscores how dynamic ctDNA burden can be across the perioperative period. Importantly, the initial postoperative ctDNA sample for MRD assessment should be obtained at least 2–4 weeks after surgery to allow clearance of surgery-related cell-free DNA before interpretation [49]. Postoperative ctDNA evaluation must be interpreted in relation to clinical timing. Landmark postoperative tests are performed at least two to four weeks after surgery and aim to detect molecular residual disease prior to or during adjuvant treatment. In contrast, post-adjuvant testing is used to assess for residual disease following completion of systemic treatment. Serial surveillance is performed by the collection of multiple ctDNA samples during follow-up to detect molecular relapse. These represent distinct clinical settings and should not be interpreted interchangeably. Immediately after surgery, there will be an increased release from tissue damage and inflammation of non-tumor cell-free DNA, which will temporarily lower the concentration of circulating ctDNA and may reduce the sensitivity of assays if sampling occurs too early [7,11,14,19,27,49].

In a landmark real-world multicenter cohort study involving 295 patients with esophagogastric cancer, including 124 patients with gastric cancer, the tumor-informed Signatera assay had a postoperative sensitivity of 85.7 percent and a specificity of 95.5 percent; a postoperative surveillance sensitivity of 80 percent and a postoperative surveillance specificity of 98.3 percent were also reported [19]. Because this cohort included both gastric and gastroesophageal junction adenocarcinomas, the reported results should be interpreted as evidence from a broader esophagogastric population rather than gastric cancer alone. In addition, postoperative ctDNA positivity in patients who experienced pathological complete or near-complete responses conferred a hazard ratio of 37.6 for relapse relative to patients whose ctDNA was negative; a median lead time of 78 days until radiographic recurrence was also determined [19]. The lead time provided by preclinical detection of recurrence is a hallmark feature of tumor-informed MRD detection and is directly relevant to intervention design.

### 5.2. Tumor-Agnostic Approaches

Tumor-agnostic ctDNA detection methods avoid the need for prior tissue sequencing and may offer greater scalability, although their performance in postoperative low-burden settings varies according to assay design and disease biology (Table 6). Fixed broad gene panels, epigenetic methylation analysis, genome-wide fragmentomics, and/or integrative multidimensional analyses can be utilized to detect cancer-associated signals from cfDNA without having a patient-specific mutation map [7]. Commercial hybrid-capture panels such as Guardant360 can detect single-nucleotide variants(SNVs), indels, fusions, and copy-number alterations (CNAs) in plasma and have been found useful for detecting HER2 amplifications and actionable genomic alterations in advanced gastric cancers without needing to confirm via matched tissue sequencing [12,38]. Nevertheless, due to significantly higher variant allele frequency (VAF) calling thresholds of 0.1–1%, commercial hybrid-capture panels have limitations for true MRD surveillance where circulating tumor fractions are small [7,27]. One major limitation is the inter-patient genetic diversity of gastric cancer; since no single mutation exists universally across patients, large panels must be developed to achieve adequate coverage. However, larger panels increase background noise [27]. Methylation-based tumor-agnostic approaches partially resolve this issue by targeting epigenetically stable cancer signatures. A successful example of a methylation-based tumor-agnostic approach has been assessed in cfDNA with MCTA-Seq utilizing 153 gene methylation markers. Using this method was successful in diagnosing various stages of gastric cancer and distinguishing chromosomal instability methylator phenotypes from non-CIMP tumors [48].

Additionally, a five-gene plasma methylation cluster could stratify recurrent gastric cancer patients by survival independently of conventional markers CEA and CA19-9 [15]. Fragmentomic models combining copy number variation, fragment-size profiles, and fragment-based methylation have recently been validated, achieving AUCs of 0.967 for gastric cancer detection with a sensitivity at stage I of 68.3%, substantially exceeding previous methylation-only classifiers that reported sensitivities ranging from 16.7 to 44% at early stages [64]. Although these emerging technologies demonstrate potential for population-level surveillance, their ability to assess true postoperative MRD in curative-intent contexts has yet to be evaluated prospectively (Table 7).

### 5.3. Analytical Sensitivity and Specificity

Analytical performance of ctDNA assays depends on assay design, sequence coverage and biological limitations due to very low levels of tumor shedding into blood circulation when curative surgical intervention has already been performed.

Analytical performance varies a lot among ctDNA platforms. Depending on assay design and experimental conditions, reported detection limits of variant allele frequency range from roughly 1 percent for conventional NGS to roughly 0.1 percent for highly optimized droplet digital polymerase chain reaction(ddPCR) or BEAMing assays, and even lower analytical detection thresholds have been reported for selected ultra-deep sequencing approaches such as CAPP Seq. These values describe detection capability under controlled conditions and should not be taken to be equivalent to clinical sensitivity for MRD detection post-surgery [7,27]. Because ctDNA concentrations post-surgery are usually very low, highly sensitive analytical methods and optimized sequencing strategies are generally needed for MRD assessment. Performance of assays is influenced, however, not just by sensitivity analytically but also by biological factors that affect release of ctDNA into circulation. Crucially, both biological and analytical factors can lead to false-negative ctDNA findings. In individuals with limited residual disease, diffuse-type gastric cancer, or localized peritoneal dissemination, biological false negatives arise when remaining tumor burden releases inadequate ctDNA into the circulation despite good test performance. On the other hand, technological constraints such as inadequate sequencing depth, limited panel design, low plasma DNA input, improper sampling duration, or subpar pre-analytical processing lead to analytical false negatives. Because physiologically constrained ctDNA shedding cannot be entirely overcome by increases in assay sensitivity, it is crucial to distinguish between these two pathways to minimize analytical false negatives. An important analytical confounding factor is clonal hematopoiesis of indeterminate potential (CHIP); this occurs when mutations arise in the somatic compartment of older white blood cells and appear identical to those found in tumor-derived DNA causing false positive MRD detection unless matched leukocyte DNA is utilized for removal [7,12]. Beyond analytical accuracy, false-positive ctDNA findings may also have important clinical consequences, such as incorrect attribution. The clinical implications of falsely identifying ctDNA as a positive result are potentially much greater than those related to analytical accuracy. For example, if a variant that is not derived from tumor cells is incorrectly attributed to molecular residual disease, it could lead to further unneeded imaging, an inappropriately escalated treatment plan, increased healthcare cost, and increased anxiety on behalf of the patient. It is therefore critical to employ both bioinformatic filtering techniques as well as matched leukocyte sequencing in order to minimize false positives, which will allow for an accurate assessment of postoperative molecular residual disease (MRD).

The median VAF for stage III was much less than that for metastatic gastric cancer; this illustrates a challenge of identifying circulating tumor DNA in early-stage as opposed to advanced-stage gastric cancer. A false negative is likely due to the combination of biological loss of shed circulating tumor DNA and the limitations of current assays versus the sequencing depth alone [3].

Sampling and pre-analytical factors also have a significant impact on assay performance. Plasma is generally preferable to serum; secondary high-speed centrifugation is necessary to remove white blood cell fragments, and hemolysis during sample processing can result in up to a 50-fold alteration in the reference range for the expression level of miRNAs [55].

Important performance metrics include analytical sensitivity, specificity, clinical sensitivity and specificity, and predictive values, and these should not be used interchangeably. Analytical performance measures describe an assay’s technical ability to detect defined molecular targets under controlled conditions but clinical performance also depends on biological factors such as tumor burden, ctDNA shedding, metastatic sites and patient characteristics specific to disease. Consequently high performance analytically does not necessarily translate into equivalent performance for surveillance after surgery for MRD [2,7,12,27]. Consequently, when interpreting postoperative ctDNA results, the biological context of each patient as well as assay performance characteristics should always be taken into account.

### 5.4. Landmark Studies

Research has determined the clinical course of MRD based on ctDNA in gastric cancer. A pooled analysis of 25 studies found postoperative ctDNA positivity to be strongly associated with decreased OS (HR = 3.47, *p* < 0.000001, 95% CI: 1.98–6.10) and DFS (HR = 4.14, *p* < 0.000001, 95% CI: 2.43–7.07), both stronger associations than before surgery, identifying postoperative MRD sampling as the most clinically useful time frame [65]. Yang et al. studied a cohort of 46 patients, demonstrating that ctDNA positivity was associated with poorer DFS and OS with hazard ratios of 14.78 and 7.66 respectively. The authors further noted that these results were significant because they also preceded radiographic recurrence by a mean of 6 mo [66].

Kim et al. reported similar findings in which post-surgical ctDNA was detectable for an average of 4.05 mo prior to radiographic recurrence [67]. A prospective study followed a cohort of 100 evaluable Stage II/Stage III resectable gastric adenocarcinomas for a median of 52.2 mo (NCT02887612), used strict false discovery rate (FDR) thresholds (<0.01), and utilized healthy donor matrix cross-verification to confirm ctDNA had durable prognostic utility [8]. Longitudinal ctDNA sequencing performed on advanced gastric cancer patients with peritoneal metastasis showed a discrepancy between the systemic and regional response to therapy. This finding highlighted spatial heterogeneity within plasma ctDNA [47]. Tumor-informed ctDNA testing from peritoneal lavage fluid (PLF), a primary location for recurrence after resection, detected metastatic cells in PLF with AUC of 0.93 for predicting peritoneal metastasis, and had 100% sensitivity and 84.62% specificity at an optimal threshold of 32.71 hGE/mL when combined with circulating tumor cell (CTC) detection yielded a negative predictive value of 96% [35]. Although there is increasing evidence supporting ctDNA MRD monitoring in gastric cancer, it remains investigational and cannot be recommended to influence adjuvant treatment decisions without clinical trial data providing endpoints of survival [29,68] (Table 8). Collectively, these studies consistently show ctDNA positivity post-surgery is a very important biomarker that predicts recurrence and worse survival. However important limitations should temper interpretation of results [11,13,19]. Most cohorts were conducted among East Asians using different sequencing technologies and schedules and using different definitions of positivity for ctDNA [2,7,10]. Also while molecular relapse usually occurs months before radiological recurrence it is still unclear whether earlier detection alone improves survival because no randomized trial has yet shown that guided therapeutic intervention changes long-term clinical outcomes [7,14,26,63]. Thus, current evidence supports ctDNA primarily as a prognostic and risk stratification biomarker rather than as a validated tool for making treatment decisions [2,7,14,68].

## 6. Molecular Relapse Versus Clinical Relapse

### 6.1. Liquid Biopsy Implications in Gastric Cancer Surveillance

Liquid biopsy may extend postoperative MRD assessment by enabling recognition of molecular relapse before recurrence becomes clinically or radiologically apparent. MRD refers to the persistence of hidden residual disease immediately following curative treatment, while molecular relapse means the longitudinal reappearance or persistence of ctDNA during surveillance preceding radiological or symptomatic recurrence. Although biologically connected, these represent sequential stages along the recurrence continuum rather than interchangeable ideas. This distinction is especially important when interpreting studies that look at ctDNA post-surgery: some look at baseline MRD status while others study serial molecular relapse during follow-up [7].

### 6.2. Definition of Lead Time Window

Quantification of molecular lead time over conventional imaging has been established with increasing accuracy in gastric and gastroesophageal cancer cohorts. A prospective tumor-informed ctDNA study found that molecular positivity precedes radiographic recurrence by a median of 184 days—more than 6 months—in the PLAGAST Cohort, with individual lead times ranging from 2 to 323 days [64]. Similar results have emerged from independent analyses: a median lead time of 6 months was confirmed in a localized gastric cancer trial using personalized multi-mutation deep plasma sequencing [11], while another cohort reported detection of molecular relapse an average of 4.05 months prior to conventional radiological confirmation [67]. A pooled clinical analysis determined that postoperative ctDNA positivity preceded radiographic recurrence by an average of 6 months and symptomatic clinical recurrence by 4 to 8.9 months [10]. Collectively, these studies suggest ctDNA detects recurrence molecularly earlier for many patients. However lead time advantage varies among studies because of differences among patient populations, assay methodologies and sampling strategies. Crucially, improved clinical outcomes should not be inferred from earlier molecular detection. This illustrates the possible impact of lead-time bias, in which early detection of recurrence increases the time between diagnosis and clinically noticeable illness without necessarily changing the course of gastric cancer. Therefore, whether early intervention based on ctDNA positivity can improve disease-free or overall survival determines the clinical significance of molecular lead time. This question is still being investigated in prospective interventional trials.

Mechanistically speaking, ctDNA can detect evidence of residual or recurrent disease before structural abnormalities become detectable by conventional imaging because tumor DNA is released into circulation before lesions become visible radiographically [2]. The orders of magnitude in detection sensitivity explain why molecular signals appear so much earlier than structural findings on CT/positron emission tomography imaging. Additionally, since ctDNA has a very short biological half-life and is constantly produced by replicating/dying tumor cells, it provides rapid and near-instant feedback regarding disease dynamics, allowing for capture of molecular changes in days to weeks of changes in tumor biology—far sooner than anatomical changes become fixed on imaging [29].

An unresolved important question is whether lead time at the molecular level translates into therapeutic benefit. Theory suggests earlier detection opens an opportunity to intervene when disease burden is lower but prospective validation for this has not yet been done for gastric cancer [68]. Whether disease-free survival or overall outcomes improve solely by initiating treatment based on positivity for ctDNA is not known and is one of the main goals of ongoing clinical trials guided by MRD [26].

### 6.3. Postoperative ctDNA as a Surrogate for Residual Disease

Postoperative ctDNA consistently shows a strong association with subsequent recurrence of disease across different studies. However, the evidence we have comes mainly from retrospective studies and relatively small groups, so performance as a prognostic marker should be interpreted with caution until results from larger prospective trials confirm this. In one important analysis, all patients presenting with detectable postoperative ctDNA but with totally equivocal radiologic imaging went on to develop full structural disease recurrence during follow-up [7]. Another study reported that all patients detected with ctDNA post-surgery developed recurrence of disease. Results emphasize strong prognostic value for positivity for ctDNA post-surgery but these results come from a small patient group and should not be taken as evidence of specificity that works universally for gastric cancer [69]. Collectively these results support ctDNA positivity post-surgery as a strong biomarker that increases risk of recurrence. However, current evidence is not yet strong enough to consider ctDNA a definitive predictor of recurrence for all gastric cancer patients and prospective validation in larger multicenter studies is still needed. Postoperative ctDNA positivity should not always be regarded as clinically actionable, despite it having a high prognostic value. There is currently insufficient evidence to show that treatment modification based only on ctDNA positivity improves patient outcomes, despite the fact that ctDNA is a marker of increased recurrence risk. Therefore, one of the main obstacles to the clinical application of molecular relapse monitoring continues to be the distinction between prognostic information and therapeutic decision-making.

The prognostic significance of ctDNA depends on the clinical time point at which the sample is obtained. Initial postoperative MRD assessment and post-adjuvant ctDNA evaluation represent distinct clinical landmarks and should be interpreted separately. A meta-analytic review demonstrated that postoperative or during-treatment ctDNA testing delivered significantly greater predictive impact than preoperative testing, with positivity predicting clinical failure at hazard ratios for overall survival (HR OS) = 3.69 and HR DFS = 5.72, compared to preoperative values (HR OS = 2.51 and HR DFS = 2.67) [26]. Residual ctDNA collected within three months of completion of adjuvant chemotherapy proved especially impactful, signaling increased risk of clinical recurrence with a HR of 14.99 and mortality risk = 11.88—rendering post-adjuvant ctDNA status among the most prognostically informative assessments currently available in gastric cancer surveillance. Higher quantitative thresholds for the presence of circulating tumor-specific mutations in cfDNA (per nanogram) or plasma (per milliliter), however, did not correlate with an increased risk of recurrence, indicating that it was the detection of ctDNA by itself (not the overall burden of ctDNA) which correlated most directly to the risk of recurrence [8].

### 6.4. The Concept of Molecular Relapse in Practice

It is crucial to recognize a biological state of MRD (residual disease), as opposed to an event of molecular relapse. Molecular residual disease (MRD) represents the presence of malignant cells or signals derived from a tumor after curative-intent treatment; such cancerous cells cannot be detected through typical clinical diagnostic testing procedures. However, they can be identified using highly sensitive molecular technology.

Molecular relapse can be described as the occurrence or re-occurrence of detectable circulating tumor DNA (ctDNA) at some point in time after detection of MRD, prior to clinical or radiographic evidence of disease recurrence [69]. Therefore, MRD is a biologic condition, and molecular relapse is a temporal, dynamic event that indicates the advancement of residual disease from an asymptomatic state to clinically apparent disease. A patient who tested MRD-free after surgical removal of their primary tumor could develop molecular relapse due to expansion of clonal residual tumors which would result in detectable levels of ctDNA in their blood throughout long-term follow-up. The temporal relationship among these two events emphasizes the need for serial testing for ctDNA postoperatively versus one-time testing [14,30]. The lead time from detection of ctDNA positivity in the pre-relapse window to radiographic recurrence was 78 days in this study. Thus, longitudinal serial surveillance will add substantial value over one-point postoperative testing [30].

This dynamic nature of molecular relapse argues strongly for serial rather than one-time testing. The kinetics of variant allele frequency (VAF) of recurrently mutated driver genes in the case of a ctDNA have been validated as being better than the total cfDNA load or blood-based Tumor mutational burden (TMB)in tracking the real-time progression or regression of the tumor, and thus to map the evolutionary history of the disease with a fidelity of 83.3%.

Serial VAF profiling can identify resistant clones and capture clonal relapse well before it progresses enough to be seen by either imaging or serum biomarkers. The need for prospective studies to establish evidence-based surveillance schedules is highlighted by the fact that the ideal frequency, duration, and timing of serial postoperative ctDNA monitoring have not yet been established. It provides a molecular correlation of treatment response which precedes radiographic confirmation [3].

### 6.5. Beyond ctDNA: Emerging Molecular Signals and the Peritoneal Space

The following biomarkers represent compartment-specific liquid biopsy approaches that complement plasma ctDNA in selected anatomical settings and should be considered distinct from routine postoperative plasma MRD surveillance.

While plasma ctDNA has proven to be the most valid modality for detecting molecular relapse, recent data suggest that additional liquid biopsy analytes may provide increased surveillance capabilities to different anatomical spaces and patterns of recurrence. Peritoneal lavage fluid (PLF) is a sampling method obtained intraoperatively rather than a conventional liquid biopsy performed during follow-up after surgery. For gastric cancer, PLF gives direct access to the peritoneal space where free tumor cells and nucleic acids derived from tumors may still be present even if plasma ctDNA is negative. Several studies have shown that positivity for intraoperative ctDNA in PLF predicts subsequent recurrence in peritoneum and can improve intraoperative risk stratification. However because collection of PLF requires an invasive surgical procedure it should be considered a complementary tool for intraoperative assessment rather than a substitute for serial monitoring of ctDNA in plasma during surveillance after surgery [35]. There have also been studies demonstrating that pre-resection PLF circulating free DNA positivity provided strong predictive performance for progression-free survival(PFS)in peritoneal metastasis, with a hazard ratio of 93.11 [36]. A recently established mRNA-based risk stratification model demonstrated its ability to identify a high risk of peritoneal recurrence up to sixteen months before clinical detection by abdominal CT or PET [70]. In cases of advanced disease, acquired resistance mutations found in ctDNA reappeared between two to eight cycles before radiographic progression in patients with HER2-positive gastroesophageal adenocarcinoma, further expanding the concept of molecular before clinical progression to include the context of treatment-resistant disease. These observations illustrate the value of liquid biopsy for monitoring treatment response and resistance evolution in metastatic disease rather than postoperative molecular surveillance [40].

In summary, the evidence from each study indicates that molecular relapse is a reproducible, clinically relevant occurrence in gastric cancer that reliably precedes clinical reality by many months. The lead time allowed by ctDNA and other liquid biopsy analytes offers a time frame of potential biological vulnerability during which disease burden is low, treatment options are broader and interventions (i.e., intensified surveillance, early systemic therapy or metastasis directed treatments) offer more theoretical benefits. Prospective interventional trials are required to determine whether the molecular lead time provided by liquid biopsy translates into improved survival outcomes. One of the major unanswered questions is how to implement liquid biopsy-guided management of gastric cancer. Intervention thresholds are another unsolved problem. There are currently no widely recognized standards that specify when ctDNA positivity should result in increased surveillance, treatment escalation, or repeat sampling confirmation. It is unclear if a single positive postoperative outcome is enough to change treatment or if persistent positivity needs to be shown first. Therefore, before ctDNA-guided surveillance can be regularly incorporated into clinical practice, standardized intervention thresholds must be established.

## 7. Discussion

### 7.1. Clinical Applications

Liquid biopsy shows great potential throughout the entire management of gastric cancer but clinical evidence varies a lot among indications. MRD detection post-surgery and longitudinal molecular surveillance currently represent the most mature clinical applications using ctDNA [7]. On the other hand, response monitoring for advanced disease, early cancer detection screening and compartment-specific liquid biopsies are still investigational and should be considered separately rather than extrapolated for surveillance after surgery [23].

While conventional postoperative surveillance has been shown to be woefully inadequate, it is well-established that after curatively intended gastrectomies there are still around 40% of patients who experience recurrences over the next 5 years. However, using currently available diagnostic methods including CT scans, endoscopies and classical serum tumor markers (CEA and CA19-9), only about 40% of recurrent cancers can be detected [51]. This limitation can be addressed by the use of circulating tumor DNA (ctDNA). Patients who have failed to eradicate their ctDNA following surgical intervention will exhibit extremely high recurrence hazards. Conversely, patients that remain negative for ctDNA will show improved disease-free survival [8]. In pooled meta-analysis data of Asian cohorts, ctDNA positivity demonstrated a hazard ratio of 3.58 for overall survival [26].

Aside from utilizing ctDNA, previous studies have also investigated the utility of circulating tumor cells (CTCs) measured prior to surgical intervention. These measurements were then used along with TNM staging and Ki-67 indexes to create a predictive nomogram which achieved excellent concordance index values, thereby greatly surpassing the predictive ability of TNM staging alone [52]. Additionally, panels of cell-free nucleic acids cell-free nucleic acids assessed postoperatively for various biomarkers provide better performance in detecting minimal residual disease than individual marker assessments [1]. Mutations in CBLB have been observed in 15% of patients with advanced gastric cancer compared to less than 2% of the general population; thus, these mutations correlate with a 14-fold increased risk of rapid progression or death within one year [13]. A combination of preoperative ctDNA-tumor tissue variant concordance and assessment of additional clinical parameters (such as tumor size, lymphatic invasion, vascular invasion and TNM stage) provided statistically significant association with cancer recurrence in univariate analysis; therefore, this combination represents potential benefits to both liquid and solid biopsy profiling [61].

Post-resection residual molecular aberrations represent a reliable means of selecting patients for intense adjuvant chemotherapy. Postoperative ctDNA-positive status directs assignment to aggressive adjuvant treatments while avoiding excessive toxicities in molecular responders [19]. Persistent ctDNA-negative status may support scientific justification for de-intensifying adjuvant therapies for this patient group pending future prospective validation [14].

The EXPLORING trial (NCT05494060) utilizes this concept. This randomized phase II study assesses whether adding anlotinib and penpulimab to the capecitabine plus oxaplatin (XELOX) regimen enhances disease-free survival from an estimated 12 to 24 months in postoperative ctDNA-positive stage III patients. Alternatively, all other postoperative ctDNA-negative patients will be treated with standard regimens [71]. Furthermore, in HER2-positive disease, the application of trastuzumab deruxtecan in patients with ctDNA *ERBB2* copy numbers exceeding 6.0 results in an objective response rate of 75.8%, illustrating theGFR role of liquid biopsy in facilitating targeted therapy choices without requiring repeat tissue biopsies [7]. Because these studies were conducted in metastatic disease, they are presented to illustrate therapeutic monitoring rather than postoperative MRD assessment. Liquid biopsy provides the only method for obtaining real-time pharmacodynamics information during ongoing treatment. Decreasing ctDNA variant allele fraction (VAF) levels indicate favorable pathological response to therapy whereas stable or increasing trajectories signify chemoresistance prior to radiographic evidence [64]. In metastatic populations, a decrease of >50% in maximum VAF following treatment commencement was independently correlated with improved overall survival [14]. Serial ctDNA-based detection of acquired HER2-resistance mechanisms—including HER2 amplification loss in 73% of post-trastuzumab progression cases and acquisition of *KRAS, PIK3CA, BRAF, MET* and *NF1* mutations in the remaining—can be monitored through successive plasma ctDNA testing [72].

Using serial ctDNA measurement together with routine follow-up protocols offers effective complementarity: in patients who eventually recur due to molecular instability, increases in ctDNA levels often mirror increases in levels of serum tumor markers (CA19-9, CA125 and CEA) as well as appearance of new lesions on CT imaging [13]. Thus, when used together, these two approaches offer enhanced accuracy in post-surgical surveillance. Large-scale screening initiatives would need to be established to generate sufficient numbers of ctDNA-positive patients for inclusion in randomized interventional trials [48,64].

Ascitic fluid and other regional fluids can yield additional molecular information in selected patients with compartmental disease. For example, ascites yields higher rates of actionable mutation detection compared to matched plasma in patients with metastases in the peritoneum. However, these approaches are reserved for patients who already have established disease in the peritoneum and should not be considered routine post-surgery surveillance tools. Likewise, analysis of circulating tumor DNA (ctDNA) in cerebrospinal fluid is primarily useful for a small subset of patients suspected or confirmed to have involvement of the central nervous system; this analysis may better reflect intracranial disease compared to plasma ctDNA. These compartment-specific analyses complement but do not replace systemic plasma-based liquid biopsy [7]. Similarly, in cases of CNS involvement (leptomeningeal metastasis), cerebrospinal fluid-based ctDNA analyses reveal a discordance rate of 86.7% in terms of genetic characteristics between post-CNS metastasis samples and samples obtained from the original primary tumors; thus historical tissue biopsy sampling cannot reliably predict treatment modalities required to address CNS relapse [73].

Among all of the possible liquid biopsies, the best evidence currently exists in favor of using ctDNA as a tool to detect postoperative residual disease (MRD) and to determine which patients have a high enough risk of cancer recurrence. However, the use of ctDNA to guide treatment is investigational at this time; while several prospective randomized clinical trials have demonstrated that ctDNA can be used to identify subgroups of patients who may benefit from different treatments or intensification of their treatment regimen, none of these trials have shown a clear survival advantage. While CTCs, EVs, and cell-free RNA provide valuable biological information, they are largely considered to be research tools due to a lack of standardized assays and insufficient prospective data validating them [1,7,14]. The interpretation of these results is complicated by significant variability in treatment across the studies available. The published cohorts consist of patients undergoing surgery exclusively, as well as those receiving perioperative or adjuvant chemotherapy, targeted therapies, immune checkpoint inhibitors, and interventions for advanced or metastatic conditions. Due to variations in tumor burden, ctDNA release, and treatment-associated molecular changes across these clinical scenarios, findings should not be directly applied from one therapeutic context to another. Prospective studies should therefore stratify outcomes according to disease stage, treatment exposure, and clinical setting, particularly when distinguishing postoperative MRD surveillance from treatment monitoring in advanced or metastatic disease.

### 7.2. Current Challenges

Beyond technical hurdles, another challenge is determining the optimal clinical implementation of MRD testing. Questions persist regarding ideal sampling intervals, management of transient ctDNA positivity, integration with conventional imaging, and escalation of appropriate therapy for patients who test positive for ctDNA but radiologically negative. Until standardized clinical algorithms become available, interpretation of ctDNA results should remain within frameworks of multidisciplinary decision-making or prospective clinical trials [7,14].

#### 7.2.1. Low Levels of ctDNA Shedding

Levels of ctDNA shedding are typically low in localized gastric cancer or early-stage gastric cancer; hence, it is very difficult to isolate the low abundance ctDNA tumor alleles (<0.1%) even with deep sequencing efforts [2]. In one study cohort of 20 patients, 15 patients exhibited no detectable genomic alterations in either their ctDNA or matching tumor tissues [61]. Low concentrations of ctDNA circulating systemically may make it difficult to determine if a patient with peritoneal dissemination has achieved an actual molecular response relative to a patient with visceral metastasis. This limitation is particularly relevant in peritoneal disease, where reduced vascularization and compartmentalized tumor growth may restrict ctDNA release into the systemic circulation [3,7,33].

#### 7.2.2. Lack of Standardization Across Collection/Processing Sequencing and Reporting Methods

Due to the lack of global standardization among institutions across sample collection/processing techniques, sequencing methodologies and reporting formats, comparison among studies is not possible [2]. Many centers employ different internal control RNAs (including U6 snRNA, miR-39 and beta actin), and there is no standardized requirement regarding secondary centrifugation to remove WBC debris [1]. It is difficult to find common ground among studies due to lack of standardized reference genes and varying cut-offs used in assays to compare results [26]. The amount of exosomal material extracted by the same instrument varies widely at different sites [74].

#### 7.2.3. Cost and Accessibility

Liquid biopsy technology requires expensive equipment, including Digital Droplet PCR and Whole-Genome Next-Generation Sequencing, which makes it unaffordable and therefore unavailable in many developing countries [2]. There are also issues regarding equitable access to this technology as much of the research validating this type of technology has been done on people living in East Asia; these genetic variations may differ from those of people living in other parts of the world [7,14]. Beyond the initial laboratory cost, longitudinal MRD surveillance may require repeated blood sampling, tumor sequencing, matched leukocyte analysis, ultra-deep sequencing, and specialized bioinformatic infrastructure. Cost-effectiveness therefore needs to be evaluated against potential benefits such as earlier recurrence detection, more selective imaging, and improved allocation of systemic therapies. At present, robust health-economic analyses of ctDNA-guided surveillance in gastric cancer remain limited.

#### 7.2.4. False Positives from CHIP

Germline contamination of circulating tumor DNA can be caused by clonal hematopoiesis of indeterminate potential (CHIP); CHIP causes benign mutations to occur in hematopoietic stem cells that are then released into circulation where they contaminate the DNA being analyzed for tumors [1]. Therefore, a method called white blood cell sequencing must be performed to identify CHIP-derived false positives; if this is not done a large number of false positive “tumor” variants result when analyzing for ctDNA. Therefore, studies that do not perform this analysis will result in significantly higher ctDNA detection rates compared to studies that filter out CHIP variants using matched buffy coats [61]. Importantly, false-positive molecular findings may have clinical consequences beyond analytical misclassification, including unnecessary imaging, repeated testing, inappropriate treatment escalation, increased healthcare costs, and patient anxiety.

#### 7.2.5. Need for Prospective Validation

Most current liquid biopsy studies contain very small sample sizes (often fewer than 200) and fail to control for pre-existing tumor heterogeneity [1]. As such, there exists no conclusive data regarding whether or not treatment decisions should be based on ctDNA positivity. Although several studies have demonstrated strong prognostic associations between ctDNA status and clinical outcomes, there is still insufficient evidence to establish that treatment modification based solely on ctDNA positivity improves long-term survival. Therefore, randomized interventional trials are necessary to determine whether ctDNA-guided escalation, de-escalation, or treatment switching provides clinically meaningful benefit [14].

#### 7.2.6. Psychological Implications of Molecular Relapse Detection

Earlier detection of molecular relapse may also have important psychological consequences. Patients may be informed that recurrence is biologically probable months before radiographic or symptomatic disease becomes apparent, sometimes in the absence of a validated therapeutic strategy. This period of uncertainty may increase anxiety, distress, and the psychological burden associated with repeated surveillance. Future ctDNA-guided pathways should therefore incorporate appropriate patient counseling and evaluate patient-reported outcomes alongside oncological endpoints.

### 7.3. Ongoing Trials and Future Directions

#### 7.3.1. MRD-Guided Treatment Strategies

EXPLORING (NCT05494060) is currently the most advanced model for MRD-guided therapy. Trim-and-Fill Meta-Analyses adjusted for publication bias indicate that ctDNA continues to exhibit strong, inverse associations with all measures of survival: hazard ratios = 2.02 (OS), 1.81 (DFS), and 2.59 (PFS). These findings provide a basis for determining the power required for upcoming interventional studies [26]. Additional studies including NCT06893133, NCT04000425, NCT05059444 and NCT04576858 are evaluating the use of ctDNA-MRD post-neoadjuvant therapy and curative resection on various platforms. Trastuzumab deruxtecan is additionally being studied in CTNA+ patients undergoing surgical intervention for HER2-positive disease in NCT05965479 and NCT06253650 [29].

#### 7.3.2. Combining with AI and Multi-Omics

Using machine learning to analyze a multimodal cfDNA assay combining fragment-size patterns, copy number variation, nucleosome coverage, and single-nucleotide substitution signatures achieved high Area Under Receiver Operating Curve area under the receiver operating characteristic curve values across three separate cohorts (a discovery cohort and two independent validation cohorts) [74]. Using least absolute shrinkage and selection operator and XGBoost methods, a 39-cell-free immune-related microRNA signature was identified that exhibited exceptional pan-cancer diagnostic performance with a high sensitivity for gastric cancer [24].

#### 7.3.3. Circulating Bacterial DNA, Tumor-Educated Platelets, and Serum Lipidomics

These represent some of the emerging analyte types that will expand liquid biopsy capabilities beyond Tumor-Derived DNA. Rather than reflecting solely on genomic alterations derived from tumors, circulating bacterial DNA offers insight into the microbiota-related changes potentially influencing gastric carcinogenesis and disease progression; tumor-educated platelets undergo RNA-related alterations resulting from their interaction with tumor cells and can be used as surrogate markers of cancer presence and biologic activity; serum lipidomics also identifies metabolically reprogrammed lipids during malignant transformation—thus representing a potential candidate for both the detection of cancer and assessment of risk. While these liquid biopsy-based biomarkers are currently under development in a clinical setting and have yet to be validated prospectively, they demonstrate how future multi-analyte liquid biopsy strategies will use combinations of genomic, transcriptomic, microbial and metabolite-based biomarkers to identify gastric cancer and monitor its response to therapy [75].

#### 7.3.4. Tailoring Personalized Surveillance Protocols

Ultimately, the goal of MRD-guided gastric cancer care is to replace static imaging schedules with dynamic, individually tailored liquid biopsy monitoring. The optimal prognostic sensitivity for routine practice could potentially be achieved through target combinations of *TP53* mutations and RASSF1A promoter methylation within a unified liquid panel [26]. Future architectures combining tumor-informed CtDNA, CfDNA fragmentomics, methylation, Ev-Rna derived from exosomes and ai-assisted clinical risk scoring will probably detect disease recurrence before it is clinically manifest in patients with gastric cancer [2].

### 7.4. Translational Perspective: From Research to Clinical Implementation

Liquid biopsy has emerged as one of the most promising innovations for surveillance after surgery for gastric cancer. Available technologies vary a lot clinically. Among currently available biomarkers ctDNA has accumulated the strongest evidence for detection of molecular residual disease (MRD), risk stratification for recurrence and longitudinal monitoring after curative treatment [2,7,10,14]. Assays informed by tumors currently stand out analytically because they perform better in terms of specificity and lower limits of detection for low-volume disease [7,11,19]. However, despite encouraging observational data showing that positivity for ctDNA consistently predicts recurrence months before radiological detection post-surgery, use of decisions guided by ctDNA has not yet become standard clinical practice because randomized trials showing improved survival are limited [7,14,26]. On the other hand, approaches that are agnostic to tumors and use predefined mutation panels, methylation profiling, or multi-feature cfDNA signatures offer important practical advantages such as lack of requirement for sequencing tissue and greater scalability [2,7,15]. Methods currently used for postoperative MRD detection generally show lower analytical sensitivity and need prospective validation before routine use [7,11]. Circulating tumor cells, extracellular vesicles and exosomal RNA along with cfRNA biomarkers also provide important biological insights into metastasis evolution, immune modulation and resistance to treatment but are currently largely investigational because assay standardization is poor, isolation methods are heterogeneous and clinical validation is insufficient [1,2,6,16]. Technical barriers continue to impede wide clinical adoption of liquid biopsy. These include low shedding of ctDNA in localized disease, reduced sensitivity in isolated peritoneal metastases, false positives due to clonal hematopoiesis of indeterminate potential (CHIP), variability in handling samples prior to analysis and lack of standard reporting thresholds as well as differences among sequencing platforms and bioinformatics pipelines [2,3,7,14]. Harmonization of specimen collection methods, assay methodology, analytical quality control and reporting standards will therefore be essential before liquid biopsy can be incorporated into routine algorithms following surgery [1,2,14]. Prospective studies such as PLAGAST and the ongoing EXPLORING trial along with additional MRD-directed clinical trials represent important steps toward determining whether molecular relapse detection can safely guide escalation or de-escalation of adjuvant treatment [43]. Ultimately future surveillance strategies are likely to integrate ctDNA monitoring informed by tumors along with complementary biomarkers such as methylation signatures, fragmentomics, extracellular vesicles and models assisted by artificial intelligence to improve both sensitivity and characterization for residual disease [2,6,15,24]. However, until strong prospective evidence shows better patient outcomes liquid biopsy should be considered promising as a supplement rather than replacement of established surveillance after surgery [2,7,14].

### 7.5. Limitations of This Review

This review has several limitations. First, although a structured literature search and predefined eligibility criteria were applied, the available evidence was synthesized narratively and no quantitative meta-analysis was performed. Second, substantial heterogeneity exists among the included studies with respect to disease stage, treatment setting, assay platform, analytical sensitivity, sampling timepoints, definitions of ctDNA positivity, and clinical endpoints, limiting direct comparison between studies. Third, much of the available evidence derives from retrospective or relatively small prospective cohorts, whereas randomized interventional evidence supporting ctDNA-guided treatment remains limited. In addition, several studies include mixed gastric, gastroesophageal junction, and esophageal cancer populations in which gastric cancer-specific outcomes are not always separately reported. Finally, liquid biopsy technologies are rapidly evolving, and current assay thresholds, analytical methods, and clinical applications may change as newer tumor-informed, methylation-based, fragmentomic, multi-analyte, and artificial intelligence-assisted approaches are prospectively validated.

## 8. Conclusions

Liquid biopsy has the potential to transform postoperative surveillance of gastric cancer, shifting the detection of recurrence from an imaging event to a molecularly detectable biological process. Circulating tumor DNA is currently the most clinically developed liquid biopsy assay for the assessment of molecular residual disease, stratification of recurrence risk, monitoring of response to therapy, and early detection of molecular recurrence. Available data consistently show that postoperative ctDNA positivity identifies patients at significantly increased risk of relapse and can precede imaging-based relapse by several months.

Important biological and technical challenges persist and limit routine clinical use. These include low shedding of tumor DNA in localized disease, low sensitivity for isolated peritoneal metastases, false positives related to CHIP, and a lack of standardized analytical workflows. Harmonizing assays prospectively and validating them will be essential before guided surveillance using ctDNA can be widely integrated into clinical practice.

Circulating tumor cells, extracellular vesicles, and exosomal RNA can complement ctDNA. Cell-free RNA, methylation signatures, and fragmentomics and polyomic models using artificial intelligence can also contribute. In this way, additional aspects of tumor biology and risk of recurrence can be captured. For now, liquid biopsy should be considered a promising research tool and not a definitive replacement for established surveillance strategies. Prospective interventional studies are needed to demonstrate whether detection of molecular recurrence can translate into earlier therapeutic intervention, improved survival, and personalized surveillance protocols for patients with gastric cancer.

## Figures and Tables

**Table 2:** Representative studies evaluating liquid biopsy for the early diagnosis of gastric cancer.

Study (Ref.)	Study Design	Population	Biomarker/ Analyte	Assay/ Platform	Clinical Application	Diagnostic Performance	Main Findings	Major Limitations
MCTA-Seq study [48]	Diagnostic cohort	Patients with gastric cancer and controls	cfDNA methylation	MCTA-Seq (153 methylation markers)	Early diagnosis	High diagnostic accuracy (reported in original study)	Successfully detected gastric cancer and distinguished CIMP from non-CIMP tumors	Requires further prospective validation; not designed for postoperative MRD
Five-gene methylation panel [15]	Observational cohort	Gastric cancer patients	Plasma DNA methylation	Five-gene methylation assay	Diagnosis and prognostic stratification	Independent prognostic stratification beyond CEA and CA19-9	Demonstrated prognostic value using plasma methylation signatures	Limited external validation
Guardant360 studies [39,45]	Observational studies	Advanced gastric cancer	Plasma cfDNA	Hybrid-capture NGS	Detection of actionable genomic alterations	Not designed for screening	Demonstrated detection of HER2 amplification and other genomic alterations from plasma	Advanced disease cohort; findings cannot be extrapolated to early diagnosis
Multi-omic cfDNA approaches [18,23,46]	Development studies	Mixed gastric cancer cohorts	cfDNA fragmentomics, methylation, end motifs, CNVs	AI-assisted multi-omic platforms	Early cancer detection	Diagnostic performance varies by platform	Illustrated the transition from mutation-based detection toward multidimensional cfDNA analysis	Prospective clinical validation is still required

**Table 3:** Representative studies evaluating extracellular vesicles in gastric cancer.

Study (Ref.)	Study Design	Population	Extracellular Vesicle Biomarker	Assay/ Platform	Clinical Application	Main Findings	Major Limitations
Guo et al. [53]	Prospective observational study	Patients with locally advanced gastric cancer receiving neoadjuvant chemotherapy	Exosomal lncRNA-GC1	Plasma exosome isolation followed by RT-qPCR	Monitoring response to neoadjuvant chemotherapy	Dynamic changes in exosomal lncRNA-GC1 correlated with treatment response and showed potential for early prediction of chemotherapy efficacy.	Single-center cohort; requires external validation before clinical implementation.
Dong et al. [32]	Translational laboratory and clinical study	Gastric cancer patients with and without peritoneal metastasis	Exosomal circPTBP3	Plasma exosome isolation, RNA sequencing, RT-qPCR	Prediction of peritoneal metastasis	circPTBP3 promoted mesothelial–mesenchymal transition and was significantly associated with peritoneal dissemination and poor prognosis.	Mechanistic study with limited clinical validation.
Ye et al. [20]	Translational study	Gastric cancer tissues, plasma samples and experimental models	Exosomal let-7g-5p	Exosome isolation, sequencing, functional assays	Biological mechanism of progression	Tumor-derived exosomal let-7g-5p promoted M2 macrophage polarization through the SERPINE1 pathway, facilitating tumor progression.	Mainly mechanistic; not designed as a diagnostic accuracy study.
Li et al. [21]	Experimental translational study	Gastric cancer models	Exosomal PD-L1	Molecular and functional analyses	Immune microenvironment characterization	Exosomal PD-L1 promoted expansion of myeloid-derived suppressor cells, contributing to immune evasion.	Preclinical evidence requiring clinical validation.
Zhang et al. [55]	Translational study	Gastric cancer tissues and experimental models	Exosomal miR-4745-5p/miR-3911	Exosome isolation, RNA sequencing, RT-qPCR	Mechanisms of metastasis	N2 neutrophil-derived exosomes promoted migration, invasion and metastasis through suppression of SLIT2.	Predominantly mechanistic findings.
Deng et al. [56]	Translational study	Gastric cancer tissues and experimental models	Exosomal circATP8A1	RT-qPCR, molecular assays	Tumor progression	circATP8A1 promoted macrophage M2 polarization through the miR-1-3p/STAT6 pathway, enhancing tumor progression.	Limited patient cohort; translational evidence.
Huang et al. [57]	Translational study	Gastric cancer tissues and plasma	Exosomal hsa_circ_000200	Exosome isolation, RT-qPCR	Diagnostic biomarker and metastasis	Exosomal hsa_circ_000200 promoted metastasis and demonstrated potential as a circulating biomarker.	Requires validation in larger prospective cohorts.
Shin et al. [54]	Diagnostic validation study	Multiple early-stage cancer cohorts including gastric cancer	Whole exosome spectral signature	Exosome-SERS-AI	Early cancer detection	AI-assisted Raman spectroscopy enabled highly accurate multi-cancer detection from circulating exosomes.	Not gastric cancer-specific; requires disease-specific validation.
Yang et al. [58]	Prospective biomarker study	Gastric cancer patients and healthy controls	Plasma EV small non-coding RNAs (rsRNAs/tsRNAs/piRNAs)	PANDORA-seq	Early diagnosis	Identified a triple EV small-RNA signature with excellent diagnostic performance, outperforming conventional serum biomarkers.	Recently published; external validation still needed.

**Table 4:** Representative studies evaluating circulating RNA biomarkers in gastric cancer.

Study (Ref.)	Study Design	Population	RNA Biomarker	Platform/ Assay	Clinical Application	Main Findings	Major Limitations
Okuno et al. [60]	Prospective biomarker study	Patients undergoing curative gastrectomy	Multi-RNA liquid biopsy signature	Circulating RNA expression profiling	Prediction of early recurrence	Developed a circulating RNA signature capable of identifying patients at increased risk of early postoperative recurrence following curative surgery.	Single-cohort study; requires external validation before clinical implementation.
Guo et al. [53]	Prospective observational study	Locally advanced gastric cancer receiving neoadjuvant chemotherapy	Circulating exosomal lncRNA-GC1	Plasma exosome isolation with RT-qPCR	Monitoring response to neoadjuvant chemotherapy	Dynamic changes in circulating lncRNA-GC1 correlated with treatment response and demonstrated potential for early assessment of therapeutic efficacy.	Single-center cohort; requires validation in larger prospective studies.
Yang et al. [58]	Prospective case–control study	Gastric cancer patients and healthy controls	Plasma extracellular vesicle small non-coding RNAs (miRNAs, tsRNAs, rsRNAs, piRNAs)	PANDORA-seq	Early diagnosis	Identified a three-RNA signature with high diagnostic performance that outperformed conventional serum tumor markers.	Recently published; external validation and standardization are still needed.
Wang et al. [24]	Multicenter diagnostic study	Multiple cancer cohorts including gastric cancer	Cell-free RNA transcriptome	Terminal-modification-independent cfRNA sequencing	Early cancer detection	Demonstrated that transcriptome-wide cfRNA sequencing enables sensitive early cancer detection and tumor classification using plasma samples.	Multi-cancer study; gastric cancer subgroup relatively small.
Tao et al. [17]	Prospective translational study	Gastrointestinal cancer patients	Cell-free multi-omics (cfRNA integrated with cfDNA)	Integrated multi-omics sequencing	Molecular profiling and biomarker discovery	Multi-omics analysis identified complementary cfRNA and cfDNA biomarkers, improving molecular characterization compared with single-analyte approaches.	Exploratory study; requires prospective clinical validation for routine use.

**Table 5:** Liquid biopsy analytes in gastric cancer: biological source, detectable signals, platforms, advantages and limitations.

Analyte	Biological Source	Detectable Signals	Main Platforms	Advantages	Limitations	MRD Relevance
ctDNA	Tumor cell apoptosis/necrosis	Mutations, CNAs, methylation, fragmentomics	NGS, ddPCR, CAPP-Seq, methylation assays	High specificity, longitudinal monitoring, MRD detection	Low shedding, CHIP, false negatives in peritoneal disease	Strongest current MRD evidence
CTCs	Intact tumor cells in blood	Cell morphology, proteins, genomic profile	CellSearch, SE-iFISH, microfluidics	Preserves viable cellular phenotype	Rare cells, EMT-related marker loss	Complementary prognostic value
Exosomes/EVs	Tumor and microenvironmental cells	miRNAs, lncRNAs, circRNAs, proteins, lipids	Ultracentrifugation, SEC, qRT-PCR, SERS-AI	Stable cargo, tumor–microenvironment information	Poor standardization	Useful for biology and future multi-analyte models
cfRNA/miRNAs	Tumor/stromal/immune cell RNA release	miRNAs, lncRNAs, circRNAs, tsRNAs, piRNAs	qRT-PCR, RNA-seq, PANDORA-seq	Functional tumor activity	RNA instability, normalization issues	Complementary biomarker potential

**Table 6:** Tumor-informed versus tumor-agnostic ctDNA approaches for MRD detection.

Feature	Tumor-Informed ctDNA Assays	Tumor-Agnostic ctDNA Assays
Requirement for tumor tissue	Yes	No
Method	Patient-specific mutations from tumor sequencing	Fixed panels, methylation, fragmentomics, WGS/WES-based signals
Sensitivity in low-burden MRD	Higher	Lower/moderate
Specificity	Very high when matched normal/WBC is used	Variable
Scalability	Lower	Higher
Cost/time	Higher and slower	Potentially faster
Best use	Postoperative MRD surveillance	Screening, advanced disease, tissue-unavailable cases
Main limitation	Requires tumor tissue and individualized panel	Lower sensitivity for true MRD

**Table 7:** Representative studies evaluating liquid biopsy in advanced gastric cancer.

Study (Ref.)	Study Design	Population	Clinical Application	Biomarker/ Assay	Main Findings	Major Limitations
PLAGAST (Zaanan et al.) [64]	Prospective observational study	Locally advanced gastric/GEJ adenocarcinoma receiving neoadjuvant therapy	Longitudinal treatment monitoring	Tumor-informed ctDNA (Signatera^®^)	ctDNA levels declined during treatment and postoperative ctDNA identified patients at highest recurrence risk	Primarily designed for perioperative MRD assessment rather than metastatic disease
Guardant360 studies [39,45]	Observational studies	Advanced/metastatic gastric cancer	Detection of actionable genomic alterations	Plasma cfDNA hybrid-capture NGS	Identified HER2 amplification, copy number alterations and resistance-associated genomic changes without requiring repeat tissue biopsy	Predominantly metastatic cohorts; not applicable to postoperative MRD surveillance
Commercial tumor-agnostic panel studies [12,39]	Observational studies	Advanced gastric cancer	Genomic profiling for clinic evaluation	Guardant360 and broad targeted NGS panels	Detected SNVs, indels, CNAs and HER2 amplification from plasma; useful when tissue was unavailable	Lower sensitivity for low-burden disease; not validated for MRD detection
Fragmentomics/methylation studies [18,23,46]	Development and validation studies	Gastric cancer cohorts	Comprehensive molecular profiling	Multi-omic cfDNA (fragmentomics, methylation, CNVs, AI-assisted models)	Demonstrated the ability to characterize tumor biology beyond single-gene mutation analysis	Primarily evaluated for cancer detection; limited evidence for treatment monitoring
Peritoneal metastasis longitudinal ctDNA study [47]	Longitudinal cohort	Advanced gastric cancer with peritoneal metastasis	Monitoring response to systemic therapy	Serial plasma ctDNA	Demonstrated discordance between systemic ctDNA dynamics and regional peritoneal disease, highlighting spatial heterogeneity	Reduced plasma ctDNA shedding in isolated peritoneal disease decreases assay sensitivity

**Table 8:** Landmark studies of ctDNA-based MRD and molecular relapse in gastric cancer.

(A)					
Study (Ref.)	Study Design	Population	Tumor Type	Stage	Treatment
Huffman et al. [19]	Multicenter real-world observational cohort	295 patients	Gastric, GEJ and esophageal adenocarcinoma	I–III	Curative-intent surgery ± perioperative therapy
Zaanan et al. (PLAGAST) [64]	Prospective observational cohort with retrospective ctDNA analysis	62 evaluable patients	Gastric and GEJ adenocarcinoma	Locally advanced (≥cT2 and/or cN+)	Neoadjuvant therapy followed by surgery ± adjuvant therapy
Yang et al. [66]	Prospective cohort	46 patients	Gastric adenocarcinoma	I–III	Curative surgery
Kim et al. [67]	Prospective exploratory cohort	25 patients (19 evaluable)	Gastric adenocarcinoma	Resectable disease	Curative surgery
Bian & Liu [65]	Systematic review/meta-analysis	25 studies (60 datasets)	Gastric cancer	Mixed	Variable
Bai et al. [35]	Prospective single-center cohort	High-risk patients after R0 resection	Gastric adenocarcinoma	Resectable high-risk disease	Curative surgery
(**B**)					
**Study (Ref.)**	**Assay Type**	**Sampling Time**		**MRD Positivity Criterion**	**Follow-Up**
Huffman et al. [19]	Tumor-informed personalized Signatera^®^ (16-plex mPCR-NGS)	Postoperative landmark sample and serial surveillance		≥2 patient-specific SNVs detected	Variable real-world follow-up
Zaanan et al. (PLAGAST) [64]	Tumor-informed WES + personalized Signatera^®^	Baseline, during neoadjuvant therapy, after surgery (2–12 weeks), follow-up		≥2 patient-specific SNVs above predefined threshold	Median 29 months
Yang et al. [66]	Targeted deep sequencing	Preoperative, first postoperative sample (9–48 days), serial surveillance		Detection of tumor-specific plasma mutations	Median 29.1 months
Kim et al. [67]	WGS followed by rearrangement-specific PCR/ddPCR	Preoperative and serial postoperative plasma		Detection of ≥1 patient-specific rearrangement	12 months
Bian & Liu [65]	Multiple ctDNA platforms	Pre- and postoperative		Study-specific definitions	Variable
Bai et al. [35]	Peritoneal lavage fluid ctDNA + CTC analysis	Intraoperative peritoneal lavage		PLF ctDNA cutoff 32.71 hGE/mL	2 years
(**C**)					
**Study (Ref.)**	**Detection Rate/Accuracy**	**Hazard Ratio/Prognostic Value**	**Molecular Lead Time**	**Major Findings**	**Main Limitations**
Huffman et al. [19]	Postoperative sensitivity 85.7%, specificity 95.5%; surveillance sensitivity 80.0%, specificity 98.3%	HR for relapse 37.6 in ctDNA-positive patients after pathological response	Median 78 days	Strong prediction of recurrence and shorter recurrence-free survival	Retrospective real-world cohort; mixed esophageal/GEJ/gastric population; non-uniform sampling and imaging intervals
Zaanan et al. (PLAGAST) [64]	Dynamic postoperative ctDNA detection identified patients at highest recurrence risk	RFS HR 12.94; OS HR 14.54	Median 184 days (2–323)	Persistent postoperative ctDNA independently predicted recurrence and death	Single-center observational study; retrospective assay analysis2
Yang et al. [66]	High postoperative detection associated with recurrence	DFS HR 14.78 (95% CI 7.99–61.29); OS HR 7.66 (95% CI 2.92–21.06)	Median 6 months (179 days)	ctDNA independently predicted recurrence, DFS and OS	Small si2ngle-center Chinese cohort
Kim et al. [67]	Postoperative ctDNA associated with recurrence	HR not reported; *p* = 0.029	Median 4.05 months	Molecular relapse consistently preceded imaging recurrence	Very small exploratory cohort
Bian & Liu [65]	Meta-analysis demonstrated consistent prognostic value	OS HR 3.47 (95% CI 1.98–6.10); DFS HR 4.14 (95% CI 2.43–7.07)	Not pooled	Postoperative ctDNA more prognostic than preoperative ctDNA	High heterogeneity among included studies
Bai et al. [35]	Sensitivity 100%, specificity 84.62%, NPV 96%	HR not reported	Not applicable	PLF ctDNA accurately predicted metachronous peritoneal metastasis	Regional (peritoneal lavage) sampling; not routine plasma surveillance

## Data Availability

No new data were created or analyzed in this study. Data sharing is not applicable to this article.

## Abbreviations

The following abbreviations are used in this manuscript:

GC	Gastric Cancer
MRD	Molecular Residual Disease/Minimal Residual Disease
ctDNA	Circulating Tumor DNA
cfDNA	Cell-free DNA
CTCs	Circulating Tumor Cells
EVs	Extracellular Vesicles
cfRNA	Cell-free RNA
VAF	Variant Allele Frequency
SNV	Single Nucleotide Variation
CNA	Copy Number Alteration
TMB	Tumor Mutational Burden
EMT	Epithelial–Mesenchymal Transition
MMT	Mesothelial–Mesenchymal Transition
CHIP	Clonal Hematopoiesis of Indeterminate Potential
WBC	White Blood Cell
FFPE	Formalin-Fixed Paraffin-Embedded
PLF	Peritoneal Lavage Fluid
MDSCs	Myeloid-Derived Suppressor Cells
PD-L1	Programmed Death-Ligand 1
HER2	Human Epidermal Growth Factor Receptor 2
GEJ	Gastroesophageal Junction
CNS	Central Nervous System
CSF	Cerebrospinal Fluid
LM	Leptomeningeal Metastasis
NGS	Next-Generation Sequencing
ddPCR	Digital Droplet PCR
WES	Whole-Exome Sequencing
WGS	Whole-Genome Sequencing
CAPP-Seq	Cancer Personalized Profiling by Deep Sequencing
SERS	Surface-Enhanced Raman Spectroscopy
MCTA-Seq	Methylated CpG Tandems Amplification and Sequencing
PANDORA-seq	RNA Sequencing Platform
SE-iFISH	Subtraction Enrichment and Immunostaining-Fluorescence In Situ Hybridization
EpCAM	Epithelial Cell Adhesion Molecule
CEA	Carcinoembryonic Antigen
CA19-9	Carbohydrate Antigen 19-9
CA72-4	Carbohydrate Antigen 72-4
CA125	Carbohydrate Antigen 125
miRNA/miR	MicroRNA
lncRNA	Long Non-Coding RNA
circRNA	Circular RNA
piRNA	PIWI-Interacting RNA
tsRNA	Transfer RNA-Derived Small RNA
snoRNA	Small Nucleolar RNA
HR	Hazard Ratio
OS	Overall Survival
DFS	Disease-Free Survival
PFS	Progression-Free Survival
ORR	Objective Response Rate
AUC	Area Under the Curve
AUROC	Area Under the Receiver Operating Characteristic Curve
FDR	False Discovery Rate
R0	Complete Resection (no residual tumor)
PICO	Population, Intervention, Comparator, Outcome
